# MicroRNA network regulation of developmental bone toxicity in a human embryonic stem cell osteogenic model

**DOI:** 10.1016/j.namjnl.2026.100108

**Published:** 2026-07-02

**Authors:** Ashley V. Schwartz, Desiree Williams, Michael H. Zepeda, Luisa B. Bertotto, Uduak Z. George, Nicole I. zur Nieden, Nicole R.L. Sparks

**Affiliations:** a Computational Science Research Center, San Diego State University, San Diego, CA, USA; b Department of Molecular, Cell & Systems Biology and Stem Cell Center, College of Natural and Agricultural Sciences, University of California Riverside, Riverside, CA, USA; c Department of Biology, California State University San Bernardino, San Bernardino, CA, USA; d Department of Environmental and Occupational Health, UC Irvine Joe C. Wen School of Population & Public Health, Irvine, USA; e Department of Mathematics and Statistics, San Diego State University, San Diego, CA, USA

**Keywords:** Developmental bone toxicity, MicroRNA networks, Osteogenic differentiation, Human embryonic stem cells, New approach methodologies (NAMs), Toxicogenomics

## Abstract

Developmental exposure to environmental toxicants is a cause of skeletal abnormalities. Yet the molecular mechanisms linking early exposure to impaired bone formation remain undefined. Skeletal tissues arise from both neural crest- and mesoderm-derived lineages that rely on shared osteogenic differentiation programs, suggesting that disruption of common regulatory processes may contribute to diverse skeletal outcomes. MicroRNAs (miRNAs) are key post-transcriptional regulators of gene networks and have appeared as indicators of toxicant-induced perturbation.

In this study, we examined whether developmentally relevant toxicants are associated with miRNA regulatory networks during osteogenic differentiation. Using a human embryonic stem cell (hESC)-based osteogenic differentiation model, we assessed the effects of nine toxicants spanning distinct primary mechanisms. Toxicant exposure impaired osteogenic differentiation at their IC50, as reflected by altered expression of osteogenic markers and transcriptional remodeling. Global miRNA profiling revealed dysregulation of miRNAs enriched for bone-related biological processes, including regulators of osteogenic commitment and differentiation timing.

Integrated miRNA–mRNA network analysis identified a subset of miRNAs linked to core osteogenic and lineage-associated pathways, including *RUNX2*-dependent transcription and BMP and Wnt signaling. Modulation of representative miRNAs produced osteogenic outcomes consistent with those observed following toxicant exposure and, in some cases, was associated with partial restoration of differentiation in exposed cultures.

Collectively, these findings indicate that chemically diverse developmental toxicants are associated with miRNA-mediated regulatory patterns during osteogenic differentiation. Identification of shared miRNA regulatory features provides mechanistic insight into developmental bone toxicity and supports the use of miRNA network analysis as a human-relevant endpoint for skeletal hazard identification.

## Highlights

• Developmental toxicants impair hESC osteogenic differentiation.
• An 11-miRNA signature is recurrent across toxicant exposures.
• Bone-associated miRNAs exhibit altered regulatory connectivity during toxicant exposure.
• High-centrality miRNA hubs target *RUNX2*-, *DLX5/6*-, and *SOX9*-associated networks.
• miRNA network analysis supports skeletal hazard identification in a human stem cell model.

## Graphical abstract

**Figure:**
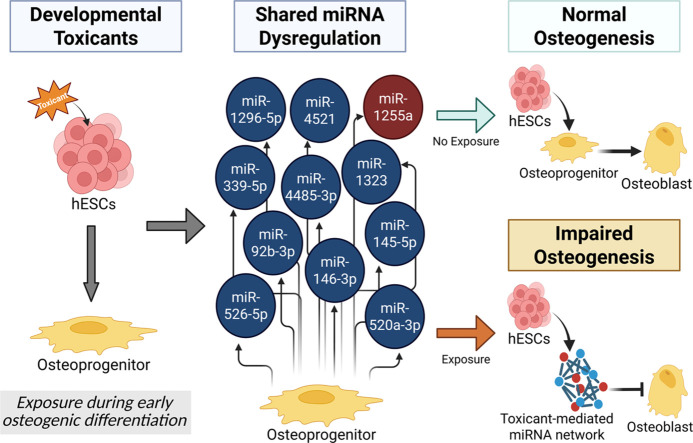

## 1 Introduction

Developmental bone toxicity remains a significant public health and regulatory concern, as disruption of bone formation during embryogenesis can result in permanent structural defects affecting craniofacial morphology, axial patterning, and appendicular growth (Akintoye et al., 2024; Ang et al., 2022; Berendsen and Olsen, 2015; Colares Neto and Alves, 2024). Although a subset of skeletal malformations arises from inherited genetic disorders, developmental exposure to environmental chemicals is an established contributor to adverse skeletal outcomes (Huang et al., 2021; Iwobi and Sparks, 2023; Knudsen et al., 2021). Importantly, chemicals with diverse molecular initiating events can produce overlapping skeletal phenotypes (Huang et al., 2021; Iwobi and Sparks, 2023; Knudsen et al., 2021), suggesting disruption of shared developmental programs governing osteogenic differentiation rather than uniform interference with upstream patterning cues.

Developmental bone toxicity is often viewed as a consequence of overt cytotoxicity or loss of osteogenic cells; however, proper skeletal development depends not only on cell survival but on the coordinated balance of bone formation and resorption (Al‐Bari and Al Mamun, 2020; Yahara et al., 2022). Bone homeostasis is governed by tightly regulated interactions between osteoblast-mediated matrix deposition and osteoclast-mediated resorption, processes that are established early during development and remain coupled throughout life (Al‐Bari and Al Mamun, 2020; Yahara et al., 2022). Disruption of osteoblast differentiation, maturation, or function, independent of cell death, can therefore shift this balance and predispose developing skeletal tissues to impaired mineralization, abnormal patterning, and long-term structural fragility (El-Gazzar and Högler, 2021; Marie, 2015). Because osteoblast differentiation provides the foundation upon which bone remodeling is later established (Bolamperti et al., 2022), early perturbation of osteogenic regulatory programs represents a critical vulnerability point for developmental bone toxicity, even in the absence of direct effects on osteoclast lineage commitment.

Skeletal development arises from multiple embryonic lineages (Galea et al., 2021; Hojo et al., 2024). Neural crest–derived progenitors contribute predominantly to the craniofacial skeleton (Newgreen and McKeown, 2005; Trainor and Krumlauf, 2000), whereas mesoderm-derived progenitors give rise to the axial and appendicular skeleton (Hojo et al., 2024; Tani et al., 2020). Despite these distinct developmental origins, both lineages rely on shared mesenchymal stem cell development and osteogenic execution machinery, including bone morphogenetic protein (BMP), Wnt, and transforming growth factor-β (TGF-β) signaling pathways (Hojo et al., 2024) and transcriptional regulation by Runt-related transcription factor 2 (*RUNX2*) (Hojo et al., 2024) and *Osterix* (*OSX*) (Hojo et al., 2024; Nakashima et al., 2002). Perturbation of these shared regulatory programs can therefore yield lineage-specific skeletal outcomes depending on exposure timing and tissue context, while still acting through common molecular nodes detectable at early stages of differentiation.

A major challenge in developmental toxicology is that traditional phenotypic endpoints, such as gross malformations, altered bone length, or impaired mineralization, occur relatively late in development and provide limited mechanistic resolution(Marx-Stoelting et al., 2021; *Scientific Frontiers in Developmental Toxicology and Risk Assessment*, 2000). This limitation has driven increased interest in new approach methodologies (NAMs) that capture early, human-relevant molecular events predictive of adverse outcomes (Lee et al., 2022; Schmeisser et al., 2023). Pluripotent stem cell–based differentiation systems have emerged as particularly valuable NAMs for developmental toxicity assessment, as they recapitulate early lineage decisions and enable mechanistic interrogation of pathway disruption prior to irreversible phenotypic outcomes (Luz and Tokar, 2018; Piersma et al., 2022).

MicroRNAs (miRNAs), particularly those associated with bone biology and commonly referred to as osteomiRs, represent a regulatory layer well suited for incorporation into NAM-based developmental toxicity frameworks. OsteomiRs fine-tune gene expression through post-transcriptional regulation of signaling pathways involved in osteogenic lineage commitment, differentiation timing, and matrix maturation (Garcia and Delany, 2021). In skeletal biology, osteomiRs regulate osteoblast commitment, maturation, and extracellular matrix production, and dysregulation of specific osteomiRs has been linked to skeletal disorders, including osteoporosis, impaired fracture healing, and craniofacial abnormalities (Sera and zur Nieden, 2017).

Beyond skeletal systems, miRNA profiling has emerged as a sensitive and predictive endpoint across multiple domains of developmental and organ-specific toxicology. Early miRNA responses have been shown to precede transcriptomic remodeling and overt adverse outcomes in contexts including developmental neurotoxicity(Smirnova et al., 2015), hepatotoxicity (Chorley et al. 2020), nephrotoxicity (Chorley et al., 2021), and pulmonary injury (Bhaskaran et al., 2012; Rogers et al., 2020). In these systems, miRNA signatures capture early state transitions and dose-dependent perturbations that predict later adverse outcomes, supporting their use as mechanistically informative endpoints within NAMs.

Within developmental osteogenesis, these properties position osteomiRs as particularly attractive mechanistic indicators, given the strong dependence of skeletal outcomes on differentiation timing and lineage context (Hojo et al., 2024; Piersma et al., 2022). However, it remains unclear whether developmental toxicants disrupt osteogenesis through lineage-specific osteomiR responses or through partially overlapping osteomiR regulatory networks that govern osteogenic fate across neural crest- and mesoderm-derived skeletal lineages.

Previous experimental studies have demonstrated that human embryonic stem cell (hESC)–derived differentiation systems can generate osteoblast populations representative of both neural crest- and mesoderm-derived skeletal lineages and that developmental toxicants disrupt osteogenic differentiation in a lineage- and timing-dependent manner (Sparks et al., 2018; Martinez et al., 2019). Collectively, this work established pluripotent stem cell–based osteogenic models as human-relevant platforms for assessing skeletal developmental toxicity, while underscoring the need to resolve how post-transcriptional regulatory mechanisms contribute to toxicant-induced impairment of osteogenesis.

In the present study, we used a hESC osteogenic differentiation model to test whether developmentally relevant toxicants with distinct primary mechanisms converge on shared osteomiR regulatory networks that suppress osteogenic differentiation. By integrating functional differentiation endpoints with miRNA sequencing, transcriptomics, pathway enrichment, and miRNA-mRNA network analysis, we identify recurrent osteomiR signatures and high-centrality regulators linking toxicant exposure to disrupted osteogenic fate. By explicitly considering both neural crest-associated and mesoderm-associated developmental contexts, this work provides mechanistic insight into how diverse toxicants interfere with bone development and supports osteomiR network dysregulation as a predictive, human-relevant endpoint for developmental bone toxicity within NAM-based assessment frameworks.

## 2 Materials and methods

### 2.1 Human embryonic stem cell culture

H9 line (WiCell Research Institute, Madison, WI) hESCs were maintained under feeder-free conditions on Matrigel-(Corning®) coated plates in mTeSR+ medium (StemCell Technologies) at 37°C in a humidified incubator with 5% CO_2_. Colonies were passaged every 4 days using accutase (2-4 min at room temperature) to displace colonies from the plastic well. Cell morphology and viability were monitored microscopically.

### 2.2 Osteogenic differentiation and exposure

Osteogenic differentiation was induced as previously described (Sparks et al., 2018, 2022; Martinez et al., 2019). Briefly, hESCs were seeded onto Matrigel-coated plates and cultured to approximately 70% confluence. Differentiation was initiated by replacing mTeSR+ with spontaneous differentiation medium composed of Dulbecco’s Modified Eagle Medium (DMEM; Gibco), 15% FBS (Seradigm), 1% (v/v) nonessential amino acids, 50 U/mL penicillin, 50 µg/mL streptomycin, and 0.1 mM βmercaptoethanol for five days. On day five, the media was supplemented with 1.2*10-7 M 1,25α(OH)2 Vitamin D3 (VD3), 0.1 mM β-glycerophosphate, and 20.8 µg/mL ascorbic acid and fed to differentiating cultures from day five onwards.

### 2.3 Toxicant exposure

Cells were exposed from day 0 to day 7 to nine toxicants at concentrations corresponding to preliminary dose-response studies using two complementary endpoints: MTT cell viability and Arsenazo III calcium deposition (osteogenic mineralization). For each toxicant, the exposure concentration to the lower (more sensitive) half-maximum inhibitory concentration (IC50) identified across the two assays, thereby ensuring that concentrations reflected the earliest detectable impairment of either cell viability or osteogenic differentiation (Table 1). This strategy provided a standardized relevant exposure level for mechanistic comparison across chemically diverse toxicants. For 5-fluorouracil, the concentration used was consistent with that previously reported by Sparks et al. (2018). The compounds represented cytotoxicity (5FU, 5-fluorouracil), oxidative stress (H2O2, hydrogen peroxide), neural-crest–targeting (CYCLO, cyclopamine; OGM, ogremorphin; MAA, methoxyacetic acid; MENOL, triadimenol), and mesodermal-targeting (CPA, cyclophosphamide; MTX, methotrexate; VPA, valproic acid).

**Table 1:** Developmental toxicants used in this study and their reported in vivo skeletal effects, molecular mechanisms, target cell populations, and testing concentrations.

Toxicant	Concentration Used in hESC Assays	Representative In Vivo Skeletal Outcomes	Primary Mechanism/ Pathway Affected	Principal Target Cells or Developmental Processes	Key References
5-Fluorouracil (5FU)	0.00025 µg/mL	Limb reduction defects, craniofacial hypoplasia, delayed or impaired ossification in rodents and zebrafish	Antimetabolite that inhibits thymidylate synthase, leading to impaired DNA synthesis and apoptosis in proliferating embryonic cells	Rapidly dividing mesenchymal progenitors and osteoprogenitors during early osteogenesis	Naren et al., 2022; Shepard, 2010
Hydrogen peroxide (H2O2)	0.017 µg/mL	Impaired bone formation, reduced bone mineral density, and growth plate injury associated with oxidative stress	Oxidative stress caused by reactive oxygen species (ROS) with secondary mitochondrial dysfunction	Osteoblasts, osteocytes, and growth plate chondrocytes exposed to oxidative damage	Iaquinta et al., 2021; Marques-Carvalho et al., 2023
Cyclopamine (CYCLO)	41.16 µg/mL	Craniofacial malformations, limb truncations, and vertebral patterning defects	Inhibition of Smoothened receptor activity resulting in suppression of Sonic Hedgehog (SHH) signaling	Neural crest cells and paraxial mesoderm involved in craniofacial and axial skeleton formation	Chen et al., 2002; Incardona et al., 1998
Ogremorphin (OGM)	0.031 µg/mL	Neural crest cell migration defects with predicted disruption of craniofacial skeletal patterning	Pharmacological inhibition of the proton-sensing G protein–coupled receptor GPR68 (OGR1), disrupting neural crest cell migration and craniofacial patterning programs	Neural crest-derived osteochondral progenitors	Williams et al., 2024
Methoxyacetic acid (MAA)	9 µg/mL	Craniofacial defects, axial skeletal malformations, and delayed ossification in rodent embryos	Disruption of histone acetylation, mitochondrial metabolism, and differentiation programs as a toxic metabolite of glycol ethers	Neural crest cells and mesodermal progenitors during early embryonic patterning and osteogenesis	Brown et al., 1984; Ritter et al., 1985
Triadimenol (MENOL)	29.58 µg/mL	Limb and craniofacial malformations with cartilage underdevelopment	Inhibition of cytochrome P450 enzymes causing altered retinoic acid homeostasis and endocrine disruption	Neural-crest–derived osteochondral progenitors	Groppelli et al., 2005; Menegola et al., 2005
Cyclophosphamide (CPA)	10 µg/mL	Limb and craniofacial malformations, ossification delay, skeletal hypomineralization	DNA alkylation and cross linking associated with oxidative stress and apoptosis	Mesodermal osteoprogenitors and endothelial precursors in limb buds	Francis et al., 1990; Rengasamy, 2017
Methotrexate (MTX)	0.01 µg/mL	Reduced bone length, craniofacial malformations, impaired osteoblast activity	Folate antagonism resulting in inhibition of nucleotide synthesis and altered methylation reactions	Mesodermal osteoblast precursors and neural-crest progenitors	Hyoun et al., 2012; Mantilla-Rivas et al., 2020; Verberne et al., 2019
Valproic acid (VPA)	10 µg/mL	Skeletal hypoplasia, vertebral defects, and craniofacial bone malformations	Histone deacetylase (HDAC) inhibition causing altered gene expression and chromatin remodeling	Neural-crest and mesodermal osteogenic mesenchyme	Gebuijs et al., 2020; Massa et al., 2005; Paradis and Hales, 2013

Note: Table 1 summarizes the developmental toxicants used in this study, their testing concentrations, reported skeletal outcomes, primary mechanisms, and principal target cell populations. Compounds were selected to represent distinct mechanistic classes relevant to bone development. Exposure concentrations were selected from preliminary MTT viability and Arsenazo III calcium mineralization dose-response studies. For each toxicant, the lower (more sensitive) IC50 was used as the exposure concentration.

### 2.4 Functional miRNA modulation during osteogenic differentiation

To assess whether toxicant-associated miRNA dysregulation contributes functionally to impaired osteogenic differentiation, selected candidate miRNAs were experimentally modulated during hESC osteogenic differentiation using Qiagen miRCURY LNA Power Inhibitors and miRNA mimics. Candidate miRNAs were selected based on differential expression across toxicant exposures and predicted regulation of bone-related target genes. For phenocopy experiments, hESCs undergoing osteogenic differentiation were transfected on day 0 with miRNA inhibitors or mimics at a final concentration of 25 nM. Negative control oligonucleotides (25 nM) and mock-transfected cultures were included as controls. Thirteen miRNA inhibitors representing downregulated miRNAs and five miRNA mimics representing upregulated miRNAs were evaluated for their effects on osteogenic differentiation (Fig. S4A). Cells were differentiated for 20 days prior to endpoint analysis. For rescue experiments, a subset of 10 candidate miRNAs was selected based on Gene Ontology enrichment and predicted osteogenic target interactions. Cells were transfected on day 0 with LNA miRNA mimics (miR-145-5p, miR-146b-3p, miR-339-5p, miR-485-3p, miR-520a-3p, and miR-4485-3p) or Power Inhibitors (miR-372-3p, miR-581, miR-1255a, and miR-4427) at 25 nM and simultaneously exposed to representative toxicants targeting neural crest-associated development (CYCLO), mesoderm-associated development (CPA), or general cytotoxic mechanisms (5FU). Osteogenic differentiation proceeded for 20 days prior to analysis (Fig. S4B).

Calcium deposition assay: Cells were harvested in radioimmunoprecipitation assay (RIPA) buffer. Calcium deposition was quantified as previously described (Sparks et al., 2018, 2022). Measurements were normalized to total protein content using the Bio-Rad DC Protein Assay Kit II.

### 2.5 Gene expression analysis

Differentiating hESCs were exposed to the identified IC50 values of each toxicant for seven days. Cells were lysed with Lysis Buffer, and RNA isolation was performed using the NucleoSpin RNA Plus Machery-Nagel Kit. Complementary DNA (cDNA) synthesis was carried out using the iScript™ Reverse Transcription Supermix for RT-qPCR (Cat# 1708840; Bio-Rad). Quantitative PCR (qPCR) was performed in triplicate using a CFX Duet Real-Time PCR System (Bio-Rad) under the following conditions: (1) pre-denaturation at 95°C for 30 s; and (2) amplification for 40 cycles of 95°C for 3 s and 60°C for 30 s. Expression levels for each gene were calculated using the ΔCt method relative to the housekeeping gene. Relative expression was determined by the ΔΔCt method, using GAPDH as a reference gene. Primer sequences are in Supplemental Table S1.

### 2.6 miRNA and mRNA sequencing

On day 7 of the differentiation protocol, cultures were lysed in QIAzol lysis reagent, miRNA and total RNA from the same sample were extracted according to the miRNeasy and RNeasy MinElute Cleanup Kits (Qiagen). Samples were assessed for RNA quality with the Bioanalyzer 2100 (Agilent), RIN values were > 7. 100 ng of each miRNA and RNA were used as input for library preparation. For the miRNA, the libraries were constructed using NEBNext® Multiplex Small RNA Library Prep Set for Illumina (NEB, Cat No.: E7330). Initial miRNA library size selection was done on a 6% PAGE gel, visualized with Sybr Gold, at ∼140 bp. This corresponds to the adapter-ligated constructs (21 nt RNA fragments and adapter). mRNA sequencing, Ribosomal RNA was depleted using the NEBNext® Poly(A) mRNA Magnetic Isolation Module (NEB, Cat No.: E7490). The libraries were constructed using NEBNext® Ultra™ II Directional RNA Library Prep Kit for Illumina (NEB, Cat No.: E7760) (supplemental material). The sequencing metadata table and complete differentially expressed miRNAs (DEMs) and mRNAs (DEGs) results are provided in the Supplementary Material. Raw and processed RNA-seq data (FASTQ files and count matrices) were deposited in the Gene Expression Omnibus (GEO) under accession GSE324490 (miRNA) and GSE324491 (mRNA).

### 2.7 miRNA sequencing analysis

miRNA libraries were sequenced on the Illumina NextSeq 500 High Output platform (1 × 75 single-end) for an average of 22 million reads at the UCR genomics core facility. Raw FASTQ files were assessed for read quality and length using Fastqc (v0.11.7) (Andrews, 2010). Low quality bases (Q<20) and reads <18 bp were trimmed using Trimmomatic (v0.36) (Bolger et al., 2014)). Trimmed reads were aligned to the indexed human genome (GRCh38) using Subread (v1.6.2) with the following parameters: “subread-align -t 1 -i HS_index -n 35 -m 4 -M 3 -T 10 -I 0 –multiMapping -B 10” ((Liao et al., 2019)). Reads were quantified using featureCounts (Subread v1.6.2) with the miRbase release 22.1 gene annotations (hsa.gff3) with the following parameters: “featureCounts -t miRNA -g Name -O -s 1 -M”. Counts were normalized using the DESeq2 median-of-ratios method (v1.36.0), and differential expression was computed using DESeq2 (v1.36.0) (Love et al., 2014). No exogenous spike-in controls were included for small RNA normalization.

### 2.8 mRNA sequencing analysis

mRNA libraries were sequenced on the Illumina NextSeq 500 High Output platform (1 × 75 single-end) for an average of 23 million reads per sample at the UCR genomics core facility. Read quality was assessed with Fastqc (v0.11.7) (Andrews, 2010). Reads were aligned to the human genome (GRCh38) using Hisat2 (v2.1.0), and aligned files were converted and sorted using samtools (v1.10) (Kim et al., 2015; Li, 2011). Gene-level quantification was performed using featureCounts (Subread v2.0.1) package (Liao et al., 2019). Counts were normalized and differential expression was computed with DESeq2 (v1.36.0) (Love et al., 2014).

### 2.9 Bioinformatic integration

DEMs and DEGs were identified using DESeq2 (BH-adjusted p < 0.05; supplemental material). Predicted and validated miRNA–target interactions were obtained from miRNet 2.0 (miRTarBase + TarBase) (Chang and Xia, 2023). Enrichment analysis for Gene Ontology (GO) Biological Processes (Ashburner et al., 2000), Kyoto Encyclopedia of Genes and Genomes (KEGG) (Kanehisa, 2000), and Disease Ontology (DO) (Schriml et al., 2012) enrichment was performed using the ClusterProfiler R package (v4.6.0) (BH-adjusted p < 0.05) (Wu et al., 2021). Over-representation analyses were performed using the default genome-wide background in ClusterProfiler.

Initial enrichment analyses were performed using unfiltered pathway outputs. Bone-related pathways were subsequently identified using a predefined keyword-based filtering strategy combined with manual review for biological relevance. Terms were screened for keywords including: "bone", "osteoblast", "osteoclast", "ossification", "skeletal", "chondrocyte", "mineralization", "osteocyte", "wnt", "bmp", "tgf", "notch", "fgf", "runx", "development", and "differentiation." Candidate pathways were then retained based on established relevance to skeletal development, bone remodeling, osteogenic differentiation, and bone-related toxicity.

### 2.10 Network construction and bone-specific filtering

miRNA-mRNA networks were constructed for each toxicant or toxicant group (5FU; H2O2; neural crest toxicants; and mesoderm) using a bipartite structure in which miRNAs were connected exclusively to mRNA targets. Edges between miRNAs and mRNAs were retained only when the following three criteria were satisfied: (1) the miRNA was significantly dysregulated (adjusted p < 0.05); (2) the mRNA target was predicted or experimentally validated in miRNet 2.0 (Chang and Xia, 2023); and (3) the mRNA was significantly differentially expressed in the corresponding RNA-seq dataset (adjusted p < 0.05).

To focus on bone-related regulatory activity, only target mRNAs annotated to skeletal development pathways were included as described in functional enrichment analysis. Networks were generated using NetworkX (v3.2.1) (Hagberg et al., 2008), and degree-centrality was calculated for each miRNA based on the number of connected bone-related target genes. The top 10 miRNAs per toxicant group were selected for downstream visualization. Subnetworks were constructed in Cytoscape (v3.10.0) (Shannon et al., 2003) using these top miRNAs and their shared targets, retaining only targets linked to ≥5 of the top miRNAs and consistently differentially expressed across group members. Bone-focused filtering was applied *a priori* to align analyses with skeletal hazard identification objectives and to enhance biological interpretability within a developmental bone toxicity framework.

### 2.11 Statistics

All data represent mean ± SEM (n ≥ 3 independent biological replicates). Statistical significance was determined by one-way ANOVA followed by Dunnett’s test (p < 0.05).

## 3 Results

### 3.1 Toxicant exposure dysregulates early lineage and osteogenic gene expression during differentiation

To characterize early molecular events underlying toxicant-induced inhibition of osteogenesis, expression of lineage-associated and osteogenic genes was assessed by RT-qPCR at day 7 of hESC osteogenic differentiation following exposure to nine developmentally relevant toxicants. This timepoint corresponds to early lineage commitment and initiation of osteogenic programs, prior to overt ECM mineralization (Martinez et al., 2019; Sparks et al., 2022). All toxicants were tested at concentrations corresponding to the IC50 values summarized in Table 1.

**Fig. 1:**
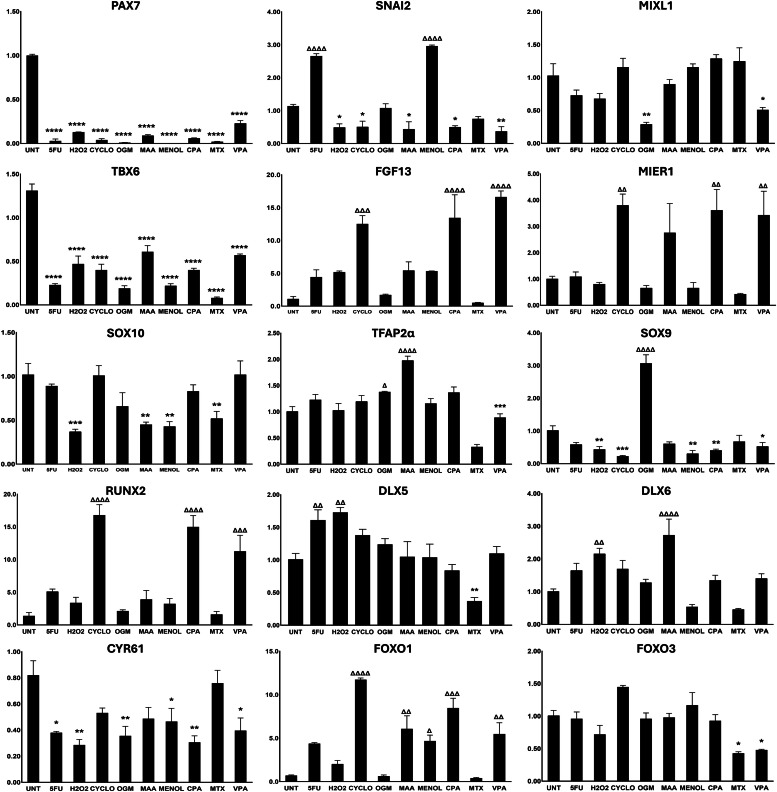
Toxicant-sensitive osteogenic markers. Quantitative PCR analysis of lineage-associated and bone-related genes at day 7 of hESC osteogenic differentiation following exposure to IC50 concentrations listed in Table 1, selected from preliminary MTT viability and Arsenazo III calcium mineralization dose-response studies. Expression values are normalized to GAPDH using the 2⁻ΔΔCt method, with vehicle control set to 1. Data are mean ± SEM (n = 3 biological replicates). Statistical significance determined by one-way ANOVA with post-hoc Dunnett test (p < 0.001). UNT, untreated; 5FU, 5-fluorouracil; H2O2, hydrogen peroxide; CYCLO, cyclopamine; OGM, ogremorphin; MAA, methoxyacetic acid; MENOL, triadimenol; CPA, cyclophosphamide; MTX, methotrexate; VPA, valproic acid.

Across toxicant exposures, genes associated with early developmental patterning and lineage commitment, including *PAX7, TBX6, SOX9, SNAI2*, and *SOX10* (Bi et al., 1999; Britsch et al., 2001; Seale et al., 2000; Shi et al., 2011; Wu et al., 2015), were frequently downregulated relative to vehicle controls (Fig. 1). These changes indicate disruption of early mesodermal and neural crest–associated transcriptional programs that normally precede orderly osteoblast differentiation. In contrast, expression of *TFAP2A*, a neural crest–associated transcription factor (Wang et al., 2011), was largely unchanged or variably regulated depending on the toxicant, suggesting selective rather than uniform disruption of neural crest–related regulatory programs.

Notably, several core pro-osteogenic transcription factors, including *RUNX2, DLX5*, and *DLX6* (Komori, 2009; Robledo et al., 2002), were maintained at control levels or exhibited increased expression at the day 7 differentiation timepoint across multiple toxicants (Fig. 1). Similarly, *FOXO1* expression was increased or not significantly altered. These patterns suggest that toxicant-induced inhibition of osteogenesis is not solely associated with broad suppression of osteogenic identity. Instead, these findings are consistent with altered coordination of osteogenic gene expression during differentiation.

The direction and magnitude of these early transcriptional changes varied among toxicants, consistent with compound-specific perturbation of distinct developmental checkpoints. However, despite these differences, all toxicants ultimately produced a shared downstream outcome of impaired osteogenic maturation and mineralization. Together, these findings suggest that developmental toxicants disrupt osteogenesis through altered coordination of lineage and osteogenic programs, rather than through simple suppression of osteogenic transcription factors.

### 3.2 Toxicant exposure induces overlapping miRNA regulatory responses during early osteogenic differentiation

To determine whether the early transcriptional dysregulation observed during osteogenic differentiation was accompanied by post-transcriptional regulatory changes, global miRNA sequencing was performed on hESC-derived osteogenic cultures at day 7 of differentiation following exposure to IC50 concentrations of each toxicant (Table 1).

**Fig. 2:**
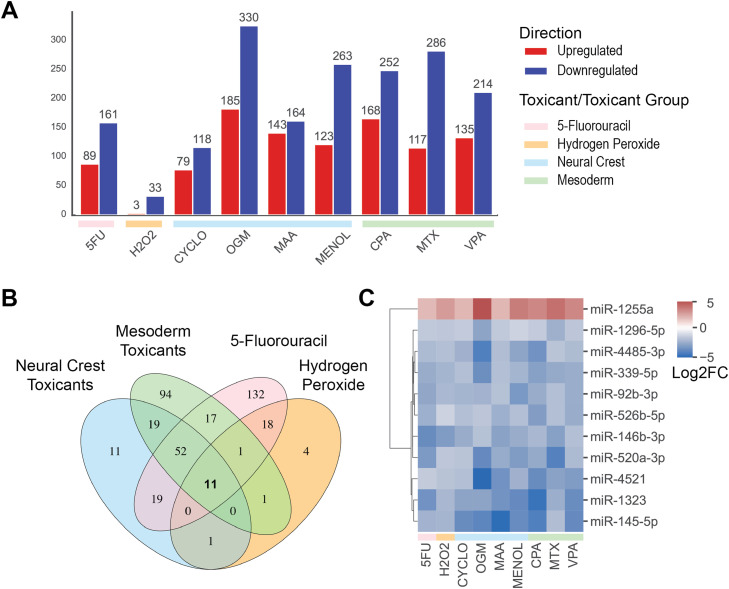
Differential regulation of miRNAs across nine developmental toxicants. Toxicants include 5-Fluorouracil (5FU), Hydrogen Peroxide (H2O2), Cyclopamine (CYCLO), Ogremorphin (OGM), Methoxyacetic acid (MAA), Triadimenol (MENOL), Cyclophosphamide (CPA), Methotrexate (MTX), and Valproic acid (VPA). (A) Bar plot showing the number of significantly upregulated and downregulated miRNAs (padj < 0.05) for each toxicant, with chemical groups annotated for known effects on the neural crest and mesoderm. (B) Venn diagram showing overlap in consistently altered miRNAs across toxicants and toxicant groups, highlighting 11 miRNAs shared across all conditions. (C) Heatmap showing the log₂ fold change of the 11 overlapping miRNAs across all treatments, illustrating patterns of up- and downregulation.

Across the nine toxicants examined, differential expression analysis identified variable numbers of significantly dysregulated miRNAs (adjusted p < 0.05), reflecting differences in toxicant class and molecular initiating events (Fig. 2A). Toxicants known to disrupt developmental signaling and differentiation programs induced broader miRNA remodeling, whereas H2O2 exposure resulted in comparatively few differentially expressed miRNAs, consistent with its primary role as an inducer of oxidative stress (Marques-Carvalho et al., 2023).

Despite this variability, intersection analysis across all nine toxicants identified an 11-miRNA set consistently dysregulated across exposure conditions (Fig. 2B, C). Of these, 10 miRNAs were downregulated and one miRNA was upregulated, indicating common miRNA regulatory responses associated with inhibition of osteogenic differentiation. Heatmap visualization demonstrated broadly consistent directionality of these miRNAs across toxicants, despite differences in the magnitude of expression changes.

**Fig. 3:**
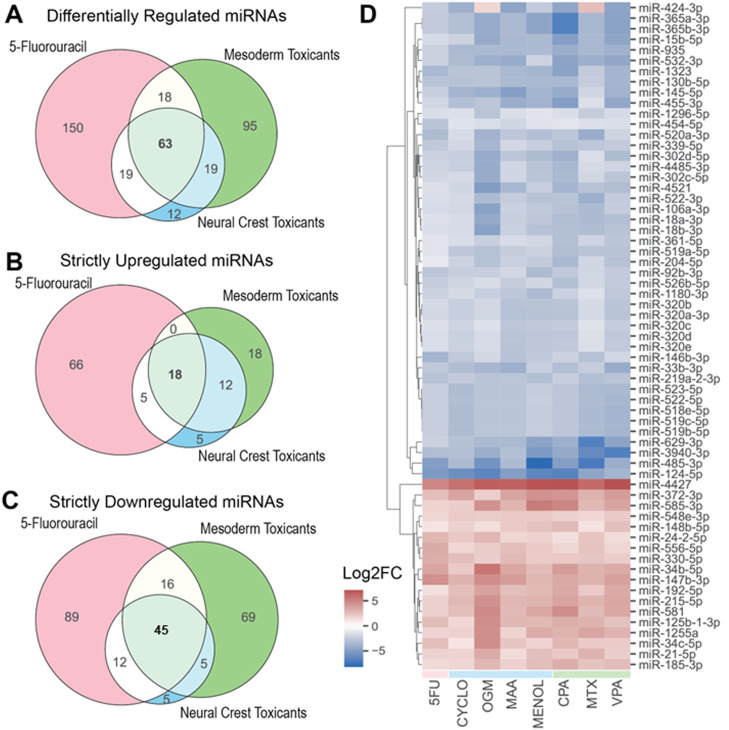
Differential regulation of miRNAs across eight developmental toxicants, excluding Hydrogen Peroxide (H2O2). Toxicants include 5-Fluorouracil (5FU), Cyclopamine (CYCLO), Ogremorphin (OGM), Methoxyacetic acid (MAA), Triadimenol (MENOL), Cyclophosphamide (CPA), Methotrexate (MTX), and Valproic acid (VPA). (A) Venn diagram showing overlap of consistently altered miRNAs (padj < 0.05) across toxicants and toxicant groups, identifying 63 miRNAs commonly regulated across all conditions with (B) showing those strictly upregulated and (C) showing those strictly downregulated. (D) Heatmap displaying the log₂ fold change of the 63 shared differentially regulated miRNAs across the eight treatments, illustrating consistent up- and downregulation patterns among neural crest toxicants, mesoderm toxicants, and 5-Fluorouracil.

Because H2O2 primarily induces generalized oxidative stress rather than direct perturbation of developmental signaling programs, and because it produced comparatively limited miRNA remodeling relative to the other toxicants, we performed a secondary sensitivity analysis excluding H2O2 to better resolve shared miRNA regulatory responses associated with toxicants that broadly disrupt developmental patterning and differentiation pathways. This analysis expanded the overlapping miRNA set from 11 miRNAs common to all toxicants to a total of 63 differentially expressed miRNAs consistently identified among the remaining developmental toxicants, comprising 18 upregulated and 45 downregulated miRNAs (Fig. 3A–D).

### 3.3 Functional validation of miRNA candidates via target pathway analysis

**Fig. 4:**
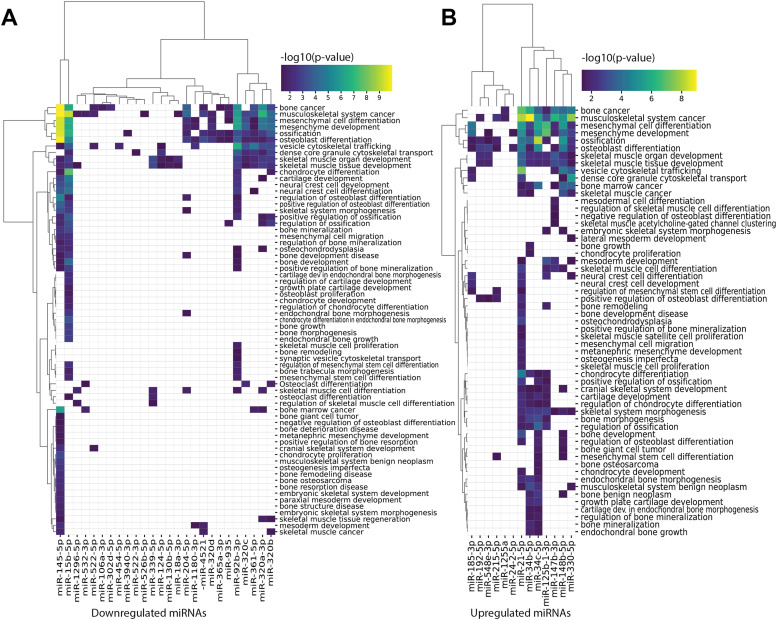
Bone-related pathway enrichment analysis of the expanded 63 candidate miRNAs (18 upregulated, 45 downregulated) shared across toxicants excluding H2O2. Heatmaps showing individual enrichment profiles (-log10 adjusted p-value) for bone-related pathways associated with 63 candidate miRNAs (45 downregulated, 18 upregulated), based on their predicted and validated gene targets. Enrichment analysis was performed using Gene Ontology Biological Process (GO), KEGG, and Disease Ontology (DO) databases. (A) Downregulated miRNAs; (B) Upregulated miRNAs. Only miRNAs with at least one significantly enriched (adjusted p < 0.05) bone-related pathway are shown.

Using this expanded miRNA set, we next assessed the biological relevance of these candidate miRNAs in the context of bone development through target-based pathway enrichment analysis (Fig. 4).

For each of the 63 DEMs, predicted and experimentally validated mRNA targets were retrieved using miRNet, which integrates interaction data from miRTarBase and TarBase. Target gene sets were subjected to pathway enrichment analysis using Gene Ontology (GO), KEGG, and Disease Ontology (DO) annotations. Bone-related pathways were identified through a combination of keyword-guided filtering and manual curation to ensure relevance to skeletal development.

Pathway enrichment analysis demonstrated that 39 of the 63 commonly dysregulated miRNAs, comprising 26 downregulated and 13 upregulated miRNAs, were significantly associated with bone-related biological processes, including osteoblast differentiation, ossification, and BMP signaling (Fig. 4). DO enrichment further linked these miRNAs to skeletal pathologies, such as osteogenesis imperfecta and skeletal system disease, establishing a functional connection between toxicant-induced miRNA dysregulation and pathways critical for bone development and skeletal homeostasis.

Several miRNAs within this bone-associated subset have been previously implicated in osteogenic regulation. The recurrent dysregulation of pro-osteogenic miRNAs, including miR-21-5p, miR-15b-5p, and miR-148b-5p (Hensley and McAlinden, 2021), is consistent with disruption of key developmental signaling pathways required for coordinated osteogenic differentiation. Both upregulated and downregulated miRNAs were represented among bone-associated regulators, indicating that toxicant exposure alters the balance and timing of miRNA-mediated regulatory control rather than uniformly suppressing osteogenic programs.

Within the more stringent 11-miRNA core set, defined as miRNAs dysregulated across all toxicants, including H2O2, 7 miRNAs exhibited significant enrichment for bone-related functions (Fig. S1). Enriched pathways included osteoblast differentiation, skeletal system development, bone cancer, and musculoskeletal system cancer, demonstrating that even the most conservative miRNA signature captures regulators linked to skeletal biology.

To further assess collective biological relevance, enrichment analysis was performed on the combined target gene sets for each miRNA group. Aggregated target analysis for the 11-miRNA set (Fig. S2) and the 63-miRNA set (Fig. S3) revealed significant enrichment for processes associated with bone development, skeletal system regulation, chondrogenesis, ossification, and bone-related diseases, including musculoskeletal cancers. These results complement the single-miRNA analyses and indicate that the candidate miRNAs, when considered as regulatory networks, influence gene programs relevant to skeletal development.

To determine whether toxicant-associated miRNA dysregulation contributes functionally to impaired osteogenic differentiation, selected candidate miRNAs were experimentally modulated during hESC osteogenic differentiation using miRNA mimics and inhibitors (Fig. S4). Modulation of individual miRNAs in the absence of toxicant exposure altered osteogenic mineralization, with several perturbations phenocopying the inhibitory effects of toxicant treatment on calcium deposition. Conversely, inverse modulation of selected miRNAs during exposure to representative toxicants partially restored osteogenic differentiation, as evidenced by increased mineral deposition relative to toxicant-treated controls. Together, these findings provide functional evidence supporting the predicted regulatory roles of candidate miRNAs and indicate that toxicant-associated miRNA dysregulation contributes mechanistically to impaired osteogenic differentiation.

### 3.4 Consolidated annotation of bone-associated miRNAs

Based on the pathway enrichment analysis shown in Fig. 4, 39 of the 63 shared differentially expressed miRNAs (26 downregulated and 13 upregulated) were significantly associated with bone-related biological processes, including osteoblast differentiation, ossification, and BMP signaling. These bone-associated miRNAs were selected for detailed annotation and are summarized in Supplemental Table S2.

Supplemental Table S2 provides a consolidated overview of miRNAs implicated in osteogenic regulation, bone differentiation, and skeletal disorders, integrating both literature-supported and newly annotated candidates from this bone-enriched subset. The table presents experimentally validated and predicted evidence supporting the roles of these miRNAs in bone formation, osteoblast differentiation, and skeletal homeostasis. miRNAs are organized in descending order of evidence strength, ranging from those with strong clinical and in vivo support (e.g., miR-21-5p, miR-145-5p, miR-204-5p) to candidate regulators identified through transcriptomic and network analyses that require further validation.

For each miRNA, Supplemental Table S2 reports the direction of dysregulation, key bone-related target genes (e.g., *RUNX2, BMP2, SMAD7, PTEN*), associated functional outcomes or bone-related disorders (including osteoporosis, osteoarthritis, fracture healing, and ossification defects), concise mechanistic interpretations, and the highest level of supporting evidence available (clinical, in vivo, in vitro, or in silico). By restricting annotation to miRNAs with demonstrated enrichment in bone-related pathways, Table S2 provides a focused, mechanistically grounded framework linking toxicant-induced miRNA dysregulation to impaired osteogenic differentiation and developmental osteotoxicity.

### 3.5 Network analysis identifies high-centrality miRNAs controlling osteogenic fate

To identify miRNAs with strong regulatory influence in toxicant-induced osteotoxicity, we constructed bone-restricted bipartite miRNA–mRNA interaction networks for individual toxicants and toxicant groups, including 5FU, H2O2, mesoderm-targeting toxicants, and neural crest–targeting toxicants. Networks integrated three data layers: (1) differentially expressed miRNAs, (2) predicted and experimentally validated miRNA–mRNA interactions from miRNet, and (3) transcript-level differential expression from RNA-seq (Figs. S5, S6), which was incorporated to refine network construction by restricting predicted miRNA targets to those that were also significantly differentially expressed in the corresponding dataset.

To focus specifically on skeletal biology, networks were restricted to include only bone-related mRNA targets, defined using a manually curated gene list derived from GO, KEGG, and DO annotations associated with bone development, osteogenesis, and skeletal disorders. miRNA–mRNA connections within the bipartite network were retained only when the mRNA target was both a predicted target of the miRNA and significantly differentially expressed following the relevant toxicant exposure.

#### 3.5.1 Degree centrality analysis reveals shared and toxicant-specific regulatory miRNAs

**Fig. 5:**
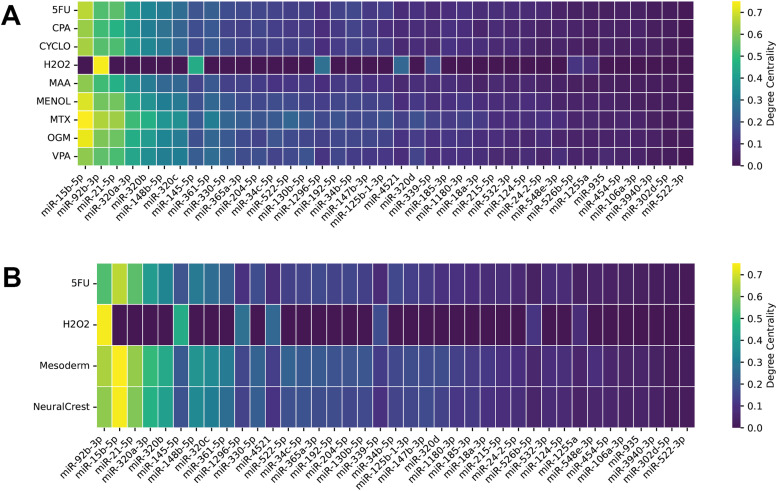
Degree centrality heatmaps of bipartite miRNA–mRNA network models for individual toxicants and toxicant groups. (A) Heatmap of degree centrality for toxicant-specific networks, including 5-Fluorouracil (5FU), Cyclopamine (CYCLO), Ogremorphin (OGM), Methoxyacetic acid (MAA), Triadimenol (MENOL), Cyclophosphamide (CPA), Methotrexate (MTX), and Valproic acid (VPA). (B) Heatmap of degree centrality for toxicant groups and general exposures: 5FU, hydrogen peroxide (H2O2), mesoderm toxicants, and neural crest toxicants. The color gradient reflects degree centrality, i.e., the number of predicted and differentially expressed bone-related mRNA targets connected to each miRNA. Higher centrality values indicate greater potential regulatory influence. miRNAs are ranked left to right in descending order of centrality.

For each network, degree centrality was calculated for individual miRNAs, representing the number of differentially expressed bone-related mRNA targets connected to each miRNA. Heatmaps of degree centrality revealed distinct connectivity patterns across toxicants and toxicant groups (Fig. 5).

Several miRNAs, including miR-15b-5p, miR-92b-3p, miR-21-5p, miR-145-5p, and members of the miR-320 family, consistently exhibited high degree centrality across multiple toxicants, indicating shared regulatory roles in osteogenic gene networks. In contrast, other miRNAs displayed elevated centrality only within specific toxicant groups, suggesting context-dependent regulatory influence. As expected, H2O2 exposure produced a sparse network with generally low miRNA centrality, consistent with its limited transcriptomic impact and primary oxidative stress–driven mechanism.

#### 3.5.2 Visualization of high-centrality miRNA regulatory networks highlights overlapping osteogenic gene modules

**Fig. 6:**
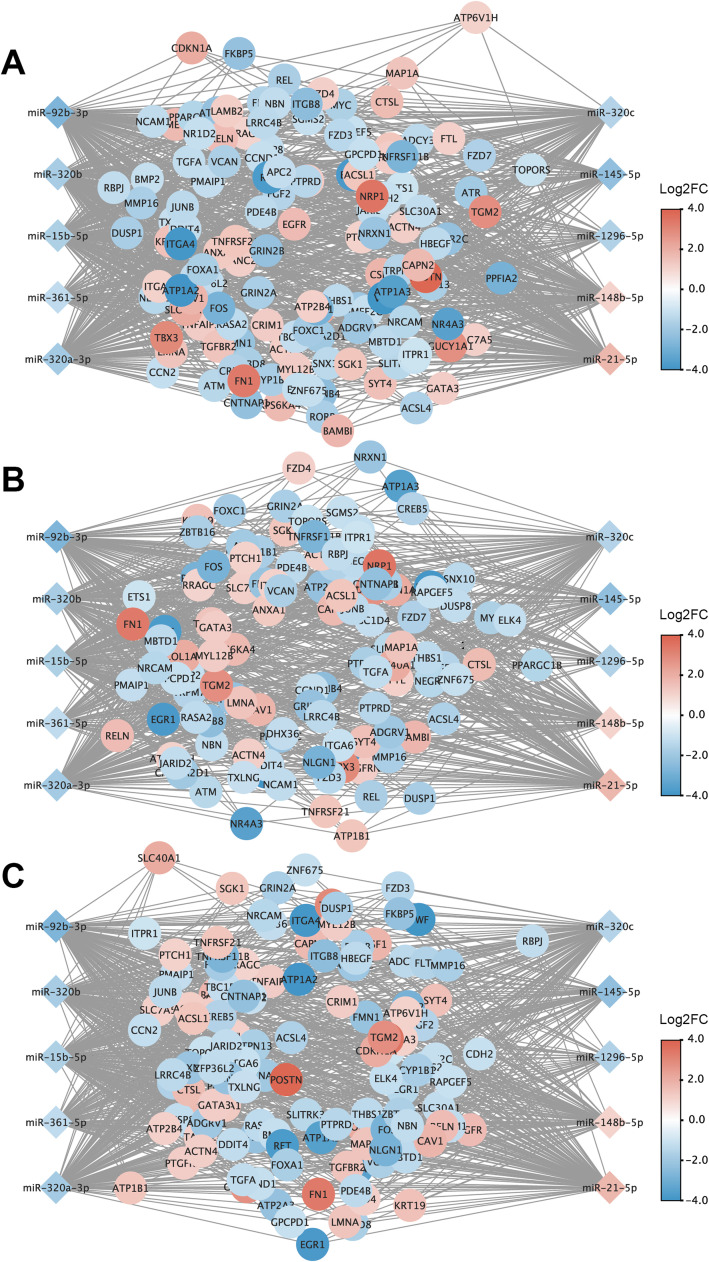
Regulatory networks of bone-related miRNAs and their differentially expressed gene targets. Bipartite network diagrams showing interactions between the top 10 miRNAs (by degree centrality; see Fig. 5B) and their predicted bone-related mRNA targets following exposure to: (A) 5-Fluorouracil (5FU), (B) mesoderm toxicants, and (C) neural crest toxicants. Nodes represent miRNAs (diamonds) and target genes (circles), with color reflecting log2 fold change (log2FC) in expression: red for consistently upregulated genes, blue for consistently downregulated genes across toxicants within a group. Edges represent predicted regulatory interactions identified using miRNet that are also significantly differentially expressed (p < 0.05). Only genes regulated by at least 5 of the top 10 miRNAs are shown to emphasize high-confidence, shared regulatory targets.

To further prioritize miRNAs with strong regulatory potential, we focused on the top 10 miRNAs by degree centrality within each toxicant group (Fig. 5B) and visualized their regulatory relationships using bipartite network diagrams (Fig. 6). Given the minimal network remodeling observed for H2O2, visualization analyses focused on 5FU, mesoderm-targeting toxicants, and neural crest–targeting toxicants.

Networks were filtered to include only bone-related genes that met two criteria: (1) predicted regulation by at least five of the top ten miRNAs, and (2) consistent differential expression across all toxicants within the group (adjusted p < 0.05). This filtering strategy emphasized shared regulatory signatures rather than toxicant-specific effects.

Across all toxicant groups, network structures were organized around *RUNX2-, DLX5/6-, SOX9-*, and *FGF13*-centered modules, reflecting overlap within the bone-focused regulatory framework applied in this analysis.

#### 3.5.3 Toxicant grouping reveals recurrent hub miRNA–mRNA network architecture across developmental mechanisms

To determine whether mechanistically distinct developmental toxicants converge on shared post-transcriptional regulatory patterns during osteogenic differentiation, toxicants were grouped according to their primary developmental mechanism and analyzed using miRNA–mRNA network integration. Although the resulting networks contained numerous differentially expressed miRNAs (Fig. 6A–C), network centrality analysis identified a restricted subset of highly connected hub miRNAs that dominated regulatory interactions within each group. Across cytotoxic (5FU), mesodermal-targeting, and neural crest–targeting toxicants, recurrent hub miRNAs, including miR-21-5p, miR-15b-5p, miR-92b-3p, miR-145-5p, and members of the miR-320 family, emerged as central regulators targeting core osteogenic and lineage-associated genes such as *RUNX2, DLX5/6*, and *SOX9*. These recurrent hub features, rather than the full set of network nodes, are summarized in Table S3 and highlight shared regulatory patterns linking diverse developmental toxicants to disrupted bone gene programs.

While Supplemental Table S3 summarizes recurrent hub miRNA–mRNA regulatory patterns within developmental toxicant groups visualized in Fig. 6, we next quantified the relative strength of miRNA network connectivity across all toxicant classes. The mesodermal group, which included VPA, MTX, and CPA, exhibited the strongest inverse miRNA–mRNA coupling (Supplemental Table S4), consistent with pronounced post-transcriptional regulation of BMP/TGF-β and Wnt/β-catenin signaling pathways governing osteoblast differentiation. In contrast, oxidative stress exposure (H2O2) showed minimal miRNA–mRNA connectivity, reinforcing its mechanistic distinction from developmental toxicants that directly perturb lineage-specifying regulatory programs. Together, these analyses indicate that toxicants disrupting developmental patterning converge on stronger and more coherent miRNA-mediated regulatory control than stress-dominated exposures.

#### 3.5.4 Target-centric transcriptomic effects of high-centrality miRNAs

**Fig. 7:**
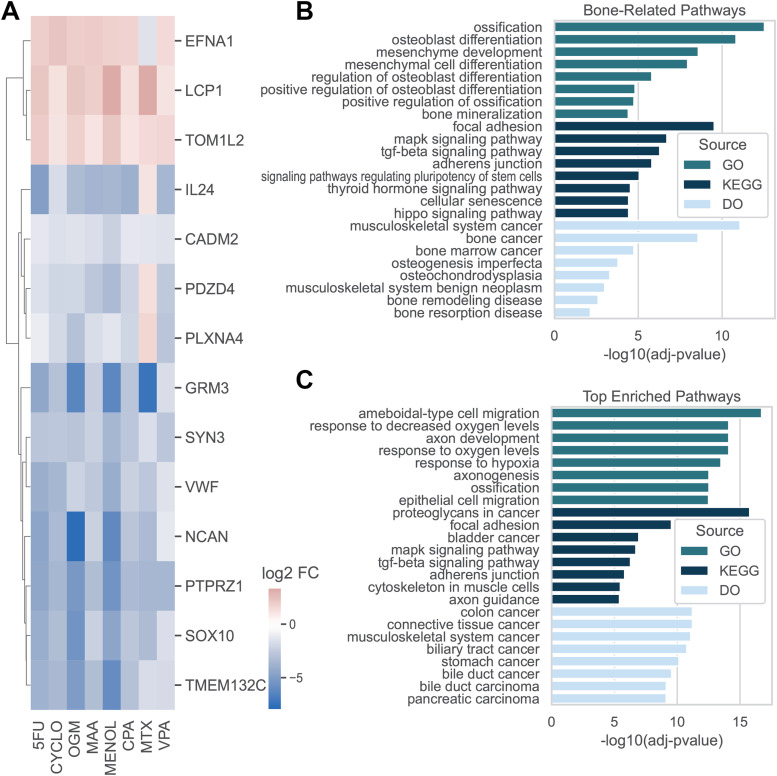
Target-centric transcriptomic responses of miR-145-5p across toxicant exposures. (A) Heatmap showing log₂ fold changes (log2FC) of genes that are predicted and/or experimentally validated targets of miR-145-5p and are significantly differentially expressed (adjusted p < 0.05) following exposure to individual toxicants. Only target genes that were significantly altered by at least one of the nine toxicants are displayed. (B) Enrichment analysis of bone-related pathways (adjusted p < 0.05) for miR-145-5p target genes that were significantly altered by any of the nine toxicants. Pathways were annotated using Gene Ontology (GO), KEGG, and Disease Ontology (DO) databases and filtered to include terms associated with skeletal development, ossification, or bone-related disease. (C) Top enriched pathways (adjusted p < 0.05) for the same miR-145-5p target gene set shown in (A), irrespective of bone association, highlighting the most statistically significant biological processes, signaling pathways, and disease annotations.

**Fig. 8:**
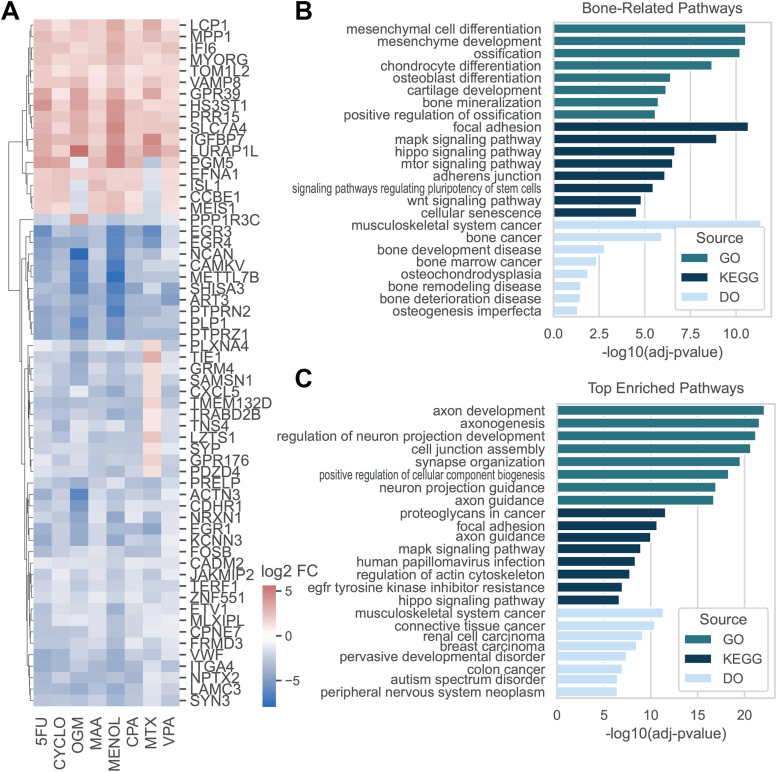
Target-centric transcriptomic responses of miR-15b-5p across toxicant exposures. (A) Heatmap showing log₂ fold changes (log2FC) of genes that are predicted and/or experimentally validated targets of miR-15b-5p and are significantly differentially expressed (adjusted p < 0.05) following exposure to individual toxicants. Only target genes that were significantly altered by at least one of the nine toxicants are displayed. (B) Bone-related pathway enrichment results (adjusted p < 0.05) for miR-15b-5p target genes that were significantly altered by any of the nine toxicants. Pathways were annotated using Gene Ontology (GO), KEGG, and Disease Ontology (DO) databases and filtered to include terms associated with skeletal development, ossification, or bone-related disease. (C) Top enriched pathways (adjusted p < 0.05) for the same miR-15b-5p target gene set shown in (A), without bone-term filtering, highlighting the most statistically significant biological processes, signaling pathways, and disease annotations.

**Fig. 9:**
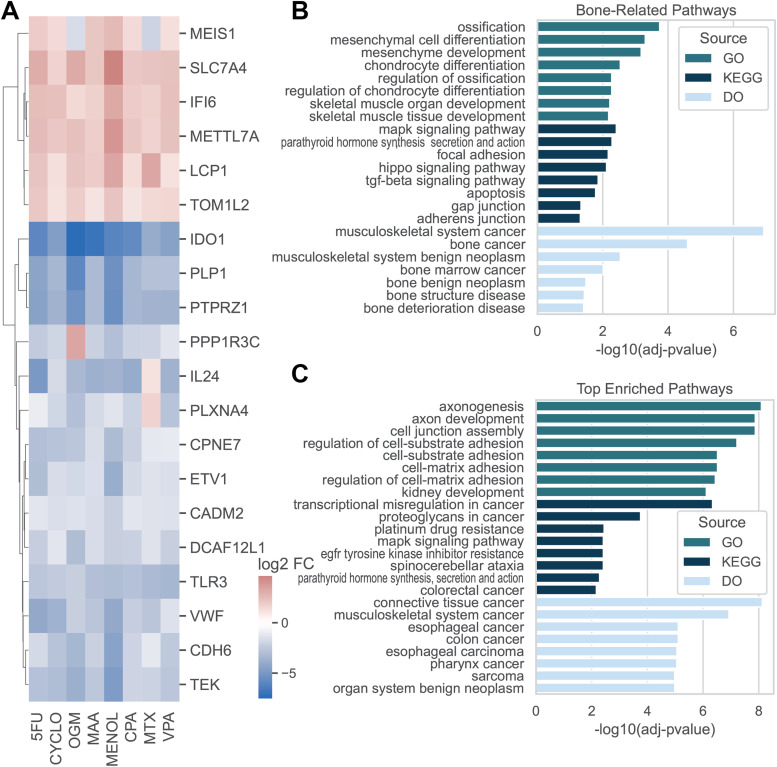
Target-centric transcriptomic responses of miR-34b-5p across toxicant exposures. (A) Heatmap showing log₂ fold changes (log2FC) of genes that are predicted and/or experimentally validated targets of miR-34b-5p and are significantly differentially expressed (adjusted p < 0.05) following exposure to individual toxicants. Only target genes that were significantly altered by at least one of the nine toxicants are displayed. (B) Bone-related pathway enrichment results (adjusted p < 0.05) for miR-34b-5p target genes that were significantly altered by any of the nine toxicants. Pathways were annotated using Gene Ontology (GO), KEGG, and Disease Ontology (DO) databases and filtered to include terms associated with skeletal development, ossification, or bone-related disease. (C) Top enriched pathways (adjusted p < 0.05) for the same miR-34b-5p target gene set shown in (A), without bone-term filtering, highlighting the most statistically significant biological processes, signaling pathways, and disease annotations.

**Fig. 10:**
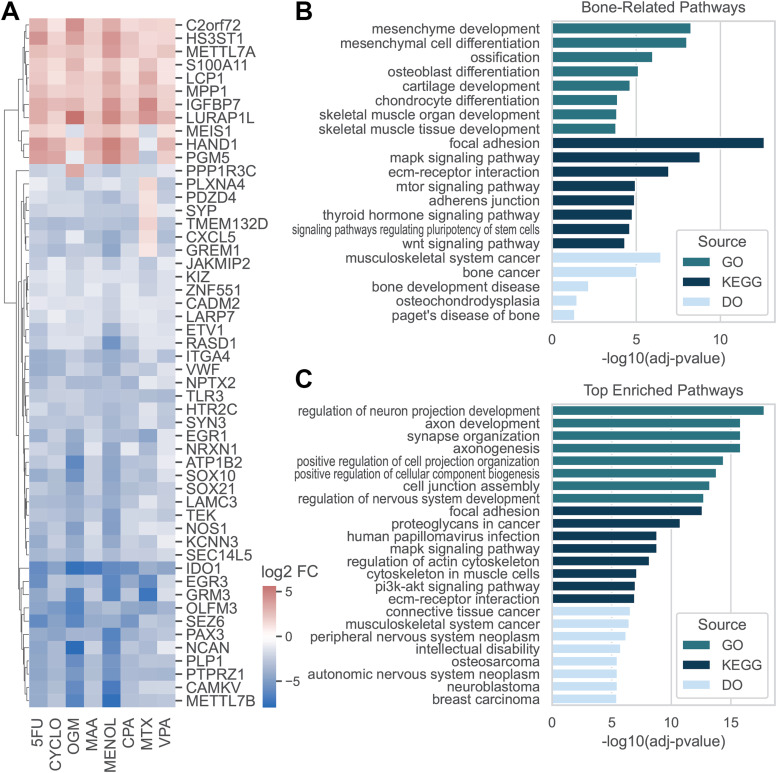
Target-centric transcriptomic responses of miR-92b-3p across toxicant exposures. (A) Heatmap showing log₂ fold changes (log2FC) of genes that are predicted and/or experimentally validated targets of miR-92b-3p and are significantly differentially expressed (adjusted p < 0.05) following exposure to individual toxicants. Only target genes that were significantly altered by at least one of the nine toxicants are displayed. (B) Bone-related pathway enrichment results (adjusted p < 0.05) for miR-92b-3p target genes that were significantly altered by any of the nine toxicants. Pathways were annotated using Gene Ontology (GO), KEGG, and Disease Ontology (DO) databases and filtered to include terms associated with skeletal development, ossification, or bone-related disease. (C) Top enriched pathways (adjusted p < 0.05) for the same miR-92b-3p target gene set shown in (A), without bone-term filtering, highlighting the most statistically significant biological processes, signaling pathways, and disease annotations.

To further evaluate the regulatory relevance of high-centrality miRNAs identified through network analysis, transcriptomic responses of their predicted and experimentally validated target genes were examined across toxicant exposures. Analyses focused on four representative miRNAs, miR-145-5p, miR-15b-5p, miR-34b-5p, and miR-92b-3p, which consistently exhibited high degree centrality across multiple toxicants and toxicant groups (Figs. 5, 6). For each miRNA, heatmaps display log₂ fold changes of target genes that were significantly differentially expressed following toxicant exposure (Fig. 7, Fig. 8, Fig. 9, Fig. 10), enabling assessment of both the directionality and coherence of downstream transcriptional responses.

Targets of miR-145-5p (Fig. 7) exhibited broadly consistent transcriptomic changes across toxicant exposures, with affected genes spanning developmental signaling, cell-cell communication, and lineage-associated processes relevant to skeletal development (Fig. 7A). The differentially expressed targets included genes implicated in neural crest function, mesenchymal differentiation, and extracellular matrix interactions, rather than being restricted to canonical osteogenic markers. Pathway enrichment analysis of miR-145-5p target genes revealed significant overrepresentation of osteoblast differentiation, ossification, and skeletal system development terms, alongside broader developmental signaling pathways (Fig. 7B). Analysis of the top enriched pathways without bone-specific filtering further demonstrated that miR-145-5p target genes were enriched for general developmental, signaling, and disease-associated processes, providing a broader context for the bone-related enrichments observed (Fig. 7C). Together, these results indicate that miR-145-5p is linked to transcriptional programs intersecting bone-related developmental processes, consistent with its high degree centrality within toxicant-responsive regulatory networks.

Similarly, miR-15b-5p target genes exhibited broadly consistent transcriptomic changes across toxicant exposures, with altered genes spanning developmental signaling, cell–cell interactions, and lineage-associated processes relevant to skeletal development (Fig. 8A). Pathway enrichment analysis of these target genes identified significant overrepresentation of bone-related terms, including mesenchymal cell differentiation, regulation of osteoblast differentiation, ossification, and skeletal system development, along with enrichment of signaling pathways such as BMP, MAPK, and Wnt signaling (Fig. 8B). Analysis of the top enriched pathways without bone-specific filtering further demonstrated that miR-15b-5p target genes were also associated with broader developmental, neuronal, and disease-related processes, providing contextual support for the bone-related enrichments observed (Fig. 8C). Together, these results indicate that miR-15b-5p is linked to transcriptional programs intersecting bone-relevant developmental pathways, consistent with its high degree centrality within toxicant-responsive regulatory networks.

For miR-34b-5p, which was upregulated across multiple toxicant conditions, target gene expression patterns were characterized by broad transcriptional shifts affecting genes involved in developmental progression, cell–cell interactions, and differentiation-associated signaling (Fig. 9A). Bone-focused enrichment analysis of miR-34b-5p target genes identified overrepresentation of ossification- and osteoblast-related processes, but also highlighted pathways associated with regulation of differentiation state and cellular organization rather than terminal osteogenic markers alone (Fig. 9B). In parallel, analysis of the top enriched pathways without bone-specific filtering revealed prominent enrichment of processes related to axonogenesis, cell–matrix adhesion, and transcriptional dysregulation in disease contexts (Fig. 9C), indicating that miR-34b-5p–associated transcriptional responses intersect broader developmental and regulatory programs. Together, these findings suggest that miR-34b-5p is linked to modulation of developmental timing and differentiation-associated transcriptional states within toxicant-responsive networks, consistent with its high degree of centrality.

Analysis of miR-92b-3p target genes revealed broadly consistent transcriptomic changes across toxicant exposures, with altered genes spanning developmental signaling, lineage-associated processes, and cell–cell and cell–matrix interaction pathways relevant to skeletal development (Fig. 10A). Bone-focused pathway enrichment analysis identified significant overrepresentation of terms related to mesenchymal development, osteoblast differentiation, ossification, and bone-associated disease categories (Fig. 10B). In contrast to the bone-filtered view, analysis of the top-enriched pathways without bone-specific filtering highlighted strong enrichment for neuronal development, axonogenesis, cytoskeletal regulation, and adhesion-related processes (Fig. 10C), indicating that miR-92b-3p–associated transcriptional responses intersect both skeletal and neurodevelopmental regulatory programs. Together, these results indicate that miR-92b-3p is linked to transcriptional programs at the interface of lineage specification and tissue organization, consistent with its high degree centrality within toxicant-responsive miRNA–mRNA networks.

Across all four miRNAs, enrichment analyses consistently identified bone-related biological processes among the most significantly affected pathways, despite variability in individual gene-level responses and toxicant-specific transcriptional signatures. Collectively, these results indicate that miRNAs prioritized by network centrality are associated with transcriptional programs closely aligned with osteogenic differentiation and skeletal development, providing target-centric support for their prominence within toxicant-responsive miRNA–mRNA networks.

#### 3.5.5 Summary of network analysis results

Collectively, these network-based analyses indicate that toxicant-associated miRNA dysregulation is organized around a limited set of high-centrality miRNAs linked to bone-related transcriptional programs across chemically and mechanistically diverse exposures. Despite distinct initiating mechanisms, toxicants were consistently associated with perturbation of shared miRNA hubs connected to *RUNX2*-, *DLX5/6*-, *SOX9*-, and *FGF13*-associated gene networks, supporting a convergent regulatory framework underlying developmental osteotoxicity.

## 4 Discussion

Developmental skeletal toxicity has traditionally been interpreted through the lens of disrupted patterning signals or overt malformations, with limited mechanistic resolution at the level of regulatory control. The findings presented here extend this framework by indicating that chemically diverse developmental toxicants are associated with overlapping post-transcriptional regulatory patterns linked to impaired osteogenic differentiation, despite distinct molecular initiating events. These overlapping regulatory responses suggest that early skeletal development contains common regulatory vulnerabilities that may be affected through multiple upstream mechanisms, leading to similar downstream functional outcomes. Notably, this overlapping regulatory response was reflected in an 11-miRNA signature identified across all toxicants examined, including H2O2 exposure. The reproducibility of this miRNA set across mechanistically distinct exposures suggests that early osteogenic differentiation engages recurrent post-transcriptional regulatory responses when developmental coordination is disrupted.

A central implication of this work is that disruption of skeletal development does not necessarily require uniform suppression of osteogenic identity. Instead, toxicant exposure appears to be associated with uncoupling of lineage progression from osteogenic differentiation, altering the coordination of regulatory programs that normally guide progenitor cells toward mature bone-forming states. Such regulatory misalignment provides a plausible explanation for how diverse toxicants produce overlapping skeletal phenotypes while maintaining heterogeneous transcriptional signatures (Guerrero-Limón et al., 2024). This interpretation aligns with developmental principles in which the sequence and context of regulatory events are as critical as their absolute activity (Graf and Enver, 2009). Because transcriptional analyses were performed at a single early differentiation timepoint (day 7), the inference of altered temporal coordination is based on cross-sectional gene expression patterns. Direct assessment of phase shifts in lineage commitment and osteogenic maturation will require time-resolved analyses. As measurements were performed on bulk cultures, selective lineage attrition or altered proliferation cannot be fully excluded as contributors to the observed transcriptional patterns.

An important consideration is how well this human embryonic stem cell osteogenic differentiation model reflects skeletal development in vivo. Although simplified, the model recapitulates the major signaling pathways that regulate early osteogenesis, including BMP, Wnt, TGF-β, and RUNX2-dependent pathways that control osteoblast specification during embryonic development. Previous studies using embryonic stem cell-derived osteogenic differentiation have also shown that this endpoint predicts developmental skeletal toxicity in vivo, supporting its use for developmental toxicity testing (de Jong et al., 2014, 2012). The toxicants examined here disrupt developmental pathways or have documented effects on skeletal development, and exposure consistently altered osteogenic differentiation together with coordinated changes in miRNA and gene expression. These molecular responses are therefore likely to represent early regulatory events associated with impaired osteogenesis. However, the model captures only early stages of osteogenic differentiation and does not reproduce the three-dimensional architecture, vascularization, immune interactions, endocrine regulation, or biomechanical influences present during skeletal development in vivo. The molecular changes identified here should therefore be viewed as mechanistic insights into early developmental bone toxicity that require further validation in vivo.

At the post-transcriptional level, miRNA-mediated regulation emerged as an important integrative layer through which disparate toxicant responses may be reconciled. Functional modulation experiments (Fig. S4) further demonstrated that perturbation of selected candidate miRNAs was sufficient to impair osteogenic mineralization and, in some contexts, partially restore differentiation during toxicant exposure. Rather than inducing broad miRNA dysregulation, developmental toxicants were associated with changes in a defined subset of high-centrality miRNAs within regulatory networks. The identification of these high-centrality miRNAs suggests that skeletal development may rely on a limited subset of regulatory miRNAs that coordinate lineage specification, developmental signaling, and osteogenic transcription. Dysregulation of these miRNAs could therefore amplify modest upstream perturbations and contribute to broader developmental consequences. Although formal statistical testing of intersection significance was not performed, the consistent directionality observed across toxicants supports reproducibility of this candidate signature within the experimental system.

Importantly, the regulatory patterns identified here were recurrent across toxicants targeting distinct embryonic lineages, indicating that neural crest– and mesoderm-derived skeletal progenitors ultimately depend on overlapping post-transcriptional control mechanisms during osteogenic differentiation. This observation challenges the assumption that lineage-specific skeletal toxicities necessarily arise from lineage-restricted regulatory logic (Wang et al., 2025). Instead, it supports a model in which lineage-specific patterning cues feed into an overlapping regulatory framework governing osteoblast differentiation and bone formation. Changes in this shared regulatory program may therefore contribute to both craniofacial and appendicular skeletal outcomes.

Differences in the strength of miRNA–mRNA coupling across toxicant classes further highlight the importance of regulatory coherence in developmental toxicity. Toxicants that interfere with developmental signaling pathways were associated with more structured and coordinated regulatory responses than stress-dominated exposures, which exhibited comparatively diffuse network organization. This distinction indicates that perturbations interfering directly with developmental regulatory programs generate more coordinated post-transcriptional remodeling than exposures dominated by generalized oxidative stress responses. This distinction supports the concept that not all perturbations impairing bone formation operate through developmental regulatory mechanisms (Wang et al., 2025) and that network-level features may help distinguish stress responses from perturbations of lineage-specifying programs (Singh et al., 2018).

From a methodological perspective, these findings underscore the value of network-based approaches for interpreting complex toxicogenomic data. Analyses focused on individual genes or pathways may overlook emergent properties arising from coordinated regulatory interactions. By contrast, identifying recurrent regulatory patterns across chemical classes provides a clearer way to interpret mechanisms despite variability in upstream toxicant action. By focusing on overlapping regulatory responses rather than individual toxicant effects, this work aligns with current efforts to develop mechanism-informed in vitro strategies for developmental hazard identification (Burbank et al., 2025; Myden et al., 2024).

The use of a human pluripotent stem cell–based osteogenic differentiation model further enhances the relevance of these observations for developmental toxicology. Such systems capture early regulatory events that are difficult to resolve in vivo and provide a human-relevant platform for integrating transcriptional and post-transcriptional responses during differentiation (Hojo et al., 2024; Luz and Tokar, 2018; Piersma et al., 2022). While in vitro models do not recapitulate tissue-level organization or biomechanical influences, they are well-suited to identifying overlapping regulatory nodes associated with developmental vulnerability (Hojo et al., 2024; Luz and Tokar, 2018; Piersma et al., 2022; Hojo et al., 2024; Luz and Tokar, 2018; Piersma et al., 2022).

The exposure concentrations used in this study were selected from preliminary dose-response studies using complementary MTT viability and Arsenazo III calcium mineralization assays. For each compound, the lower (more sensitive) IC50 from the two assays was used to provide a comparable level of osteogenic impairment across toxicants with different mechanisms of action. This approach was intended to facilitate mechanistic comparisons rather than model a specific human exposure scenario. Because nominal concentrations in static in vitro systems cannot be directly equated with tissue concentrations in vivo (Groothuis et al., 2015), the concentrations used here should not be interpreted as estimates of developmental exposure. Several compounds examined in this study, including valproic acid, methotrexate, cyclophosphamide, and 5-fluorouracil, have documented developmental skeletal effects following therapeutic exposure (Naren et al., 2022; Ornoy, 2009; Rengasamy, 2017; Verberne et al., 2019), whereas the environmental toxicants primarily represent hazard scenarios rather than typical environmental exposure levels. The findings should therefore be interpreted as identifying molecular mechanisms associated with developmental skeletal toxicity rather than defining exposure-based developmental risk. Future studies using environmentally relevant concentrations together with physiologically based in vitro-to-in vivo extrapolation will be important for quantitative risk assessment (Groothuis et al., 2015).

Several limitations warrant consideration. The regulatory interactions inferred here rely in part on predicted miRNA–target relationships, and additional experimental validation will be required to clarify the contribution of individual regulatory edges. Moreover, the differentiation system models early osteogenesis and does not encompass later stages of skeletal maturation or morphogenesis. A limitation of this study is the use of bulk differentiating cultures together with IC50-derived exposure concentrations. Because these cultures contain cells at different stages of osteogenic differentiation, toxicant exposure may alter both cellular composition and gene expression, and some of the observed changes in miRNA and mRNA abundance may therefore reflect shifts in cell populations. However, the overall patterns observed in this study are unlikely to be explained by generalized cytotoxicity alone. Osteogenic transcription factors showed variable responses across toxicants rather than suppression; similar miRNA signatures were identified despite distinct mechanisms of action, and network analyses repeatedly highlighted bone-related regulatory pathways. Together, these findings suggest that the observed molecular changes primarily reflect disrupted osteogenic differentiation, although future studies using single-cell transcriptomics and lower exposure concentrations will help distinguish changes in cellular composition from direct effects on osteogenic regulation. Future studies incorporating temporal exposure paradigms, three-dimensional differentiation systems, and in vivo validation will be important for extending these findings and evaluating their relevance to diverse skeletal outcomes. In addition, small RNA normalization was performed without exogenous spike-in controls, and therefore, global shifts in miRNA abundance cannot be fully excluded.

In summary, this study indicates that developmental skeletal toxicity is associated with recurrent miRNA regulatory signatures and overlapping high-centrality regulatory nodes rather than uniform suppression of osteogenic gene expression. Identification of recurrent high-centrality regulatory nodes provides mechanistic insight into how diverse toxicants may interfere with bone development and supports the incorporation of network-level regulatory analysis into human-relevant new approach methodologies for skeletal hazard identification.

## CRediT authorship contribution statement

**Ashley V. Schwartz:** Writing – review & editing, Visualization, Formal analysis. **Desiree Williams:** Writing – review & editing, Formal analysis. **Michael H. Zepeda:** Investigation. **Luisa B. Bertotto:** Writing – review & editing. **Uduak Z. George:** Writing – review & editing, Validation, Supervision. **Nicole I. zur Nieden:** Writing – review & editing, Validation, Supervision, Resources, Conceptualization. **Nicole R.L. Sparks:** Writing – review & editing, Writing – original draft, Validation, Supervision, Resources, Project administration, Methodology, Investigation, Funding acquisition, Formal analysis, Conceptualization.

## Declaration of competing interest

The authors declare that they have no known competing financial interests or personal relationships that could have appeared to influence the work reported in this paper.

## Data Availability

Data will be made available on request.

## Appendix Supplementary materials

Image, application 1

Image, application 2

Image, application 3

Image, application 4
