# Supplementary material for: MicroRNA network regulation of developmental bone toxicity in a human embryonic stem cell osteogenic model
Source: NAM J. 2026 Jul 2;2:100108. doi: 10.1016/j.namjnl.2026.100108 (PMC13355823; doi:10.1016/j.namjnl.2026.100108)
Supplement: Supplementary file 1 [file mmc1.docx]

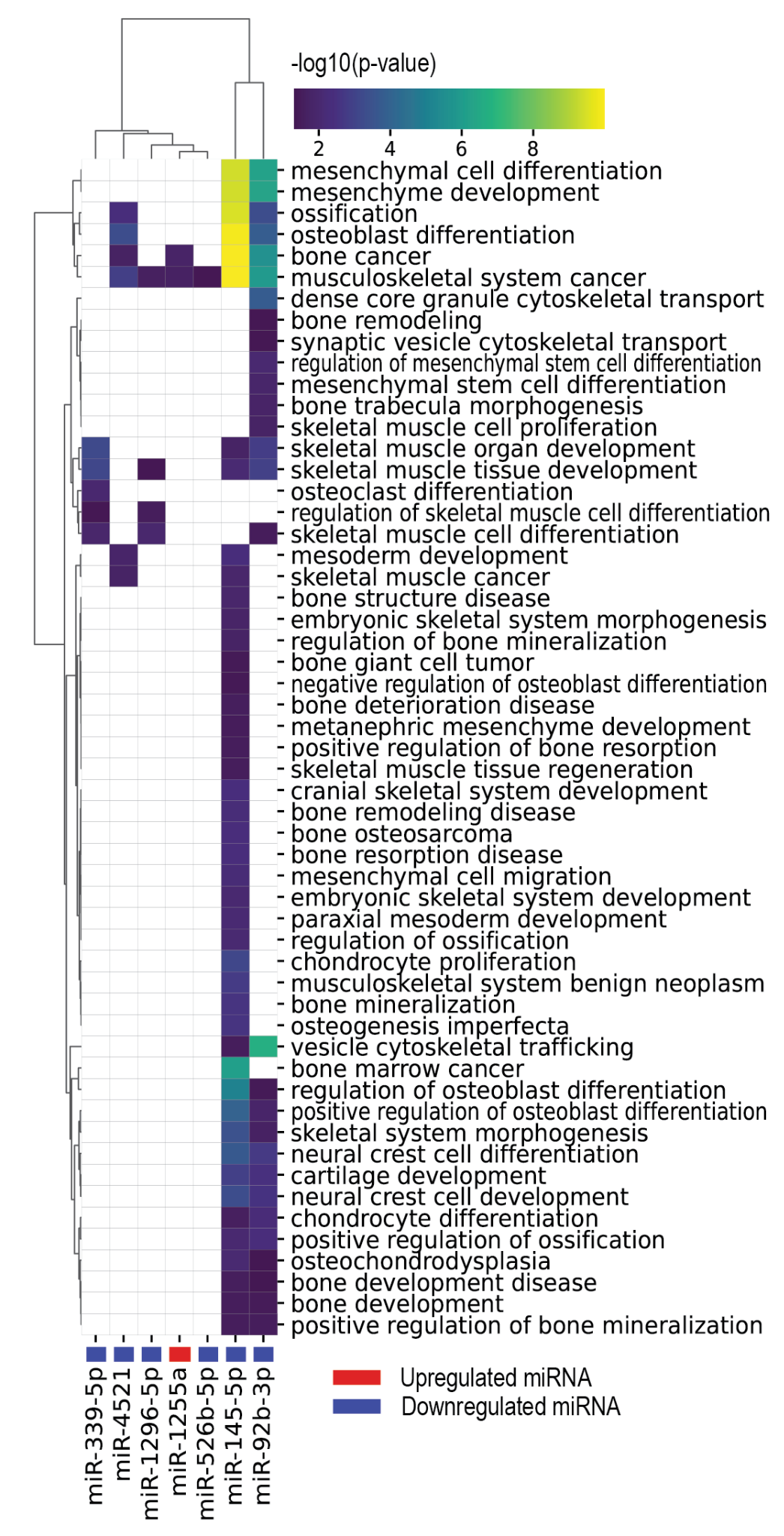


**Figure S1. Bone-related pathway enrichment analysis of the 11 candidate miRNAs (10 downregulated, 1 upregulated) shared across all 9 toxicants.**

Heatmap displaying individual enrichment profiles (-log10 adjusted p-value) for bone-related pathways associated with each of the 11 candidate miRNAs (10 downregulated, 1 upregulated), based on predicted and validated gene targets. Enrichment analysis was performed using Gene Ontology Biological Process (GO), KEGG, and Disease Ontology (DO) databases. The figure includes only the 7 miRNAs with at least one significantly enriched (adjusted p-value < 0.05) bone-related pathway.


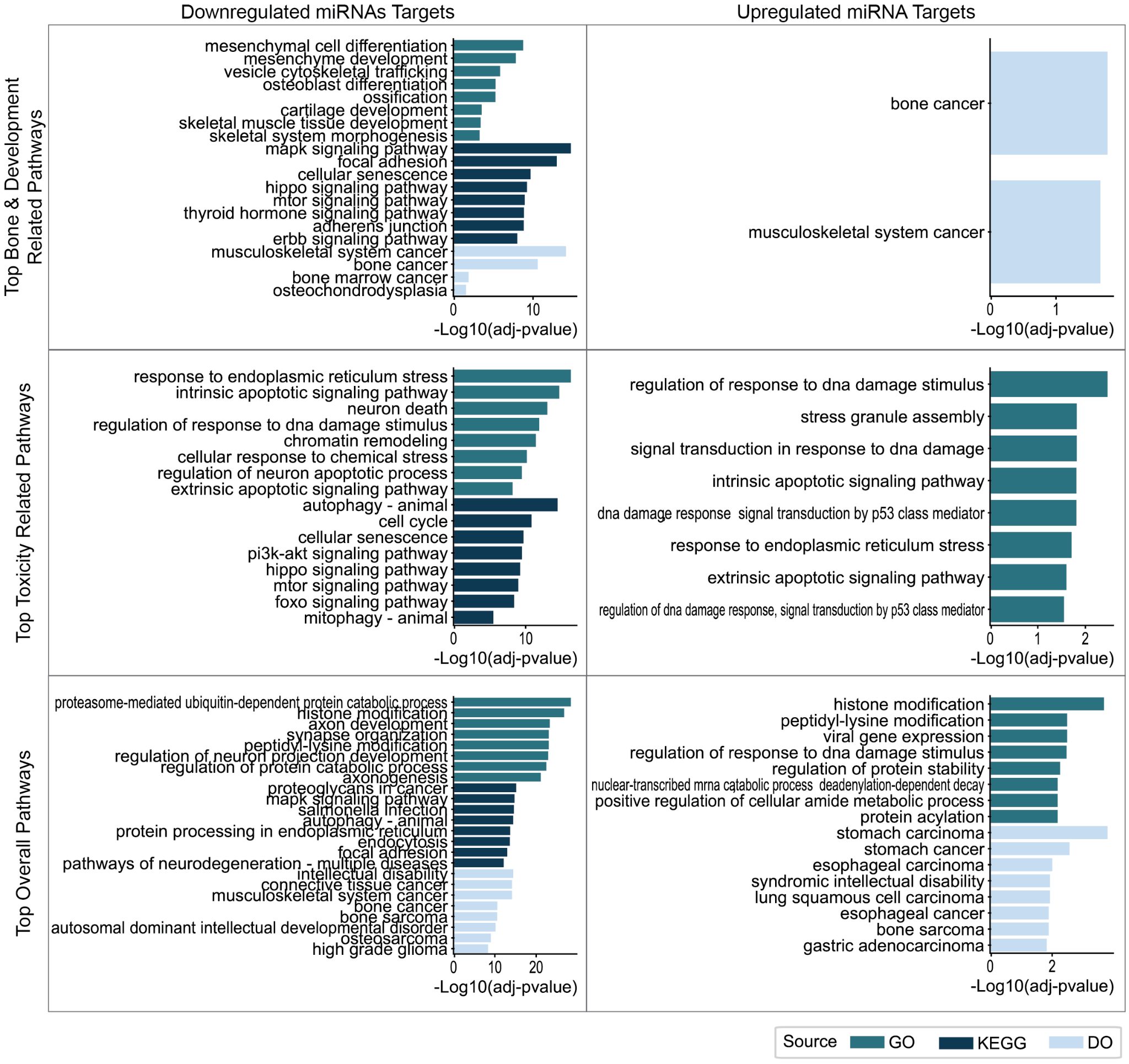


**Figure S2. Pathway enrichment analysis of the 11 candidate miRNAs (10 downregulated, 1 upregulated) shared across all 9 toxicants.** Four-panel grid displaying the top 8 enriched pathways from (-log10 adjusted p-value) from Gene Ontology Biological Process (GO), KEGG, and Disease Ontology (DO) databases. Results are shown separately for targets of upregulated and downregulated miRNAs. A subset of pathways related specifically to bone development and bone-related diseases are shown in the upper half of the grid, demonstrating consistent involvement of these miRNAs in skeletal regulatory processes. Additionally, top toxicity-related and overall pathways are shown.


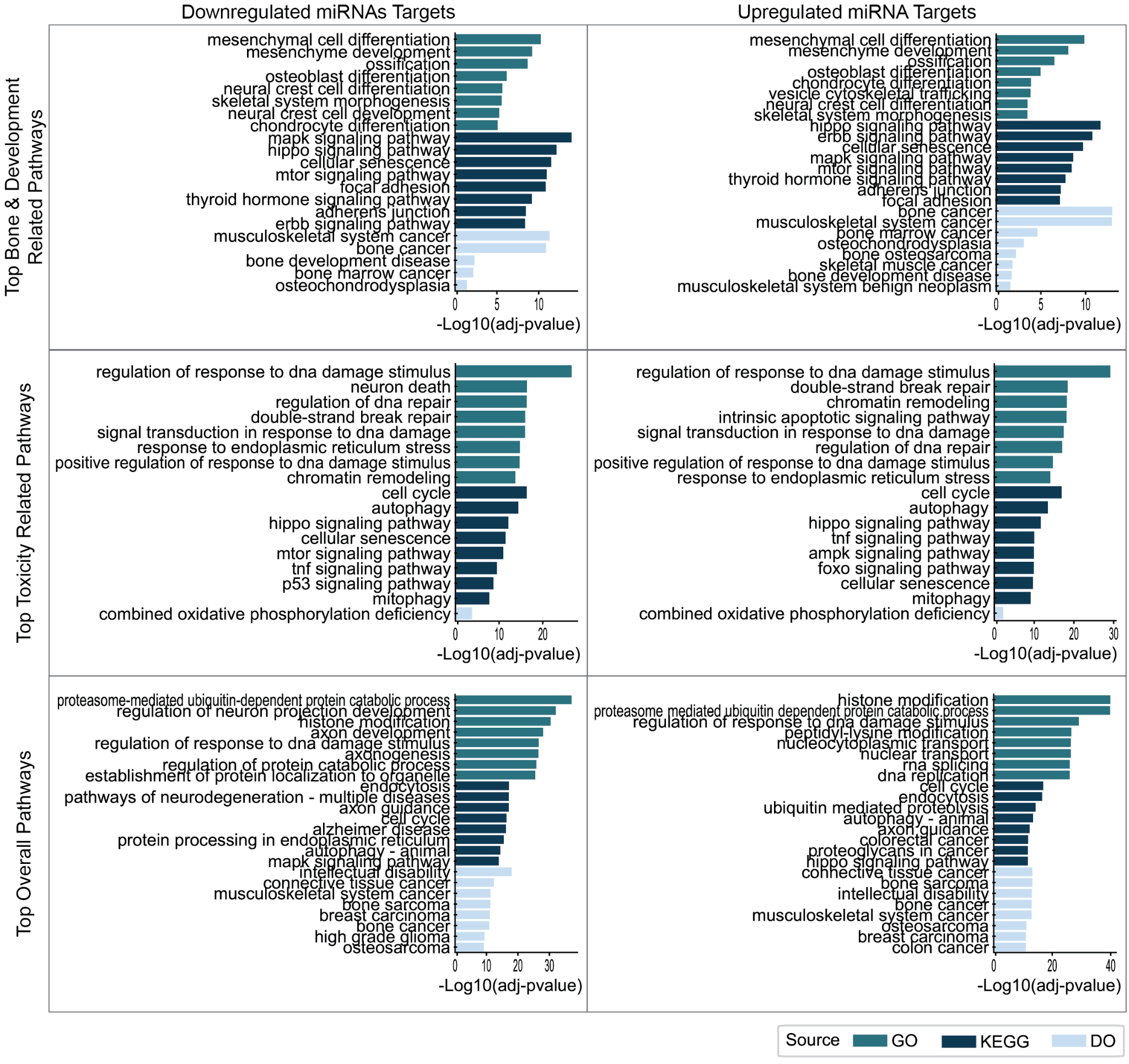


**Figure S3. Pathway enrichment analysis of the expanded 63 candidate miRNAs (18 upregulated, 45 downregulated) shared across all toxicants excluding H2O2.** Four-panel grid displaying the top 8 enriched pathways from (-log10 adjusted p-value) from Gene Ontology Biological Process (GO), KEGG, and Disease Ontology (DO) databases. Results are shown separately for targets of upregulated and downregulated miRNAs. A subset of pathways related specifically to bone development and bone-related diseases are shown in the upper half of the grid, demonstrating consistent involvement of these miRNAs in skeletal regulatory processes. Additionally, top toxicity-related and overall pathways are shown.

**A**

**B**

**Figure S4. miRNA-mediated modulation and rescue of in vitro osteogenesis.** Human embryonic stem cells (hESCs) were differentiated into osteoblasts and treated with miRNA power inhibitors or miRNA mimics to assess their effects on osteogenic differentiation. Calcium deposition was quantified as a measure of osteogenic differentiation. (A) miRNA inhibition and overexpression independently reduce osteogenic differentiation, as indicated by decreased calcium deposition relative to control. Cells were treated with Qiagen miRCURY LNA Power Inhibitors (left) or miRNA mimics (right). (B) miRNA mimics and inhibitors were applied in the presence of toxicant exposure to evaluate rescue effects. Modulation of specific miRNAs partially restores osteogenic differentiation, resulting in increased calcium deposition compared to toxicant-treated controls. Statistical significance was determined by one-way ANOVA followed by Dunnett’s multiple-comparison test. For inhibition of osteogenic differentiation: p < 0.05 (*), p < 0.005 (**), p < 0.0005 (***), p < 0.0001 (****). For rescue (increased osteogenesis): p < 0.05 (Δ), p < 0.005 (ΔΔ), p < 0.0005 (ΔΔΔ). Data are presented as mean ± SD.


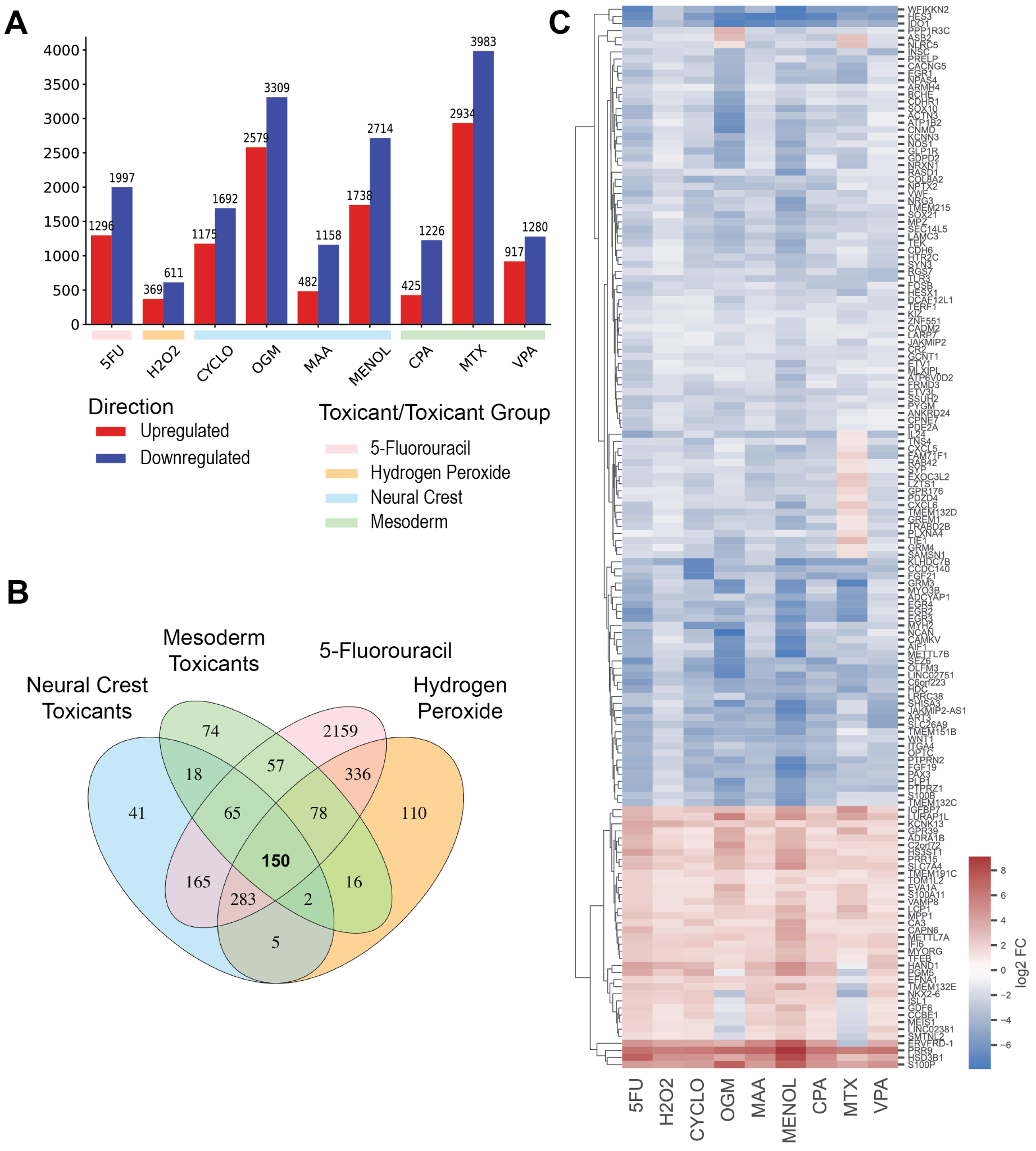


**Figure S5. Differential expression of mRNAs across nine developmental toxicants.** Toxicants include 5-Fluorouracil (5FU), Hydrogen Peroxide (H2O2), Cyclopamine (CYCLO), Ogremorphin (OGM), Methoxyacetic acid (MAA), Triadimenol (MENOL), Cyclophosphamide (CPA), Methotrexate (MTX), and Valproic acid (VPA). (A) Bar plot showing the number of significantly upregulated and downregulated mRNAs (genes) (padj < 0.05) for each toxicant, with chemical groups annotated for known effects on the neural crest and mesoderm. (B) Venn diagram showing overlap in consistently altered mRNAs across toxicants and toxicant groups, highlighting 150 mRNAs shared across all conditions. (C) Heatmap showing the log₂ fold change of the 150 overlapping mRNAs across all treatments, illustrating patterns of up- and downregulation.


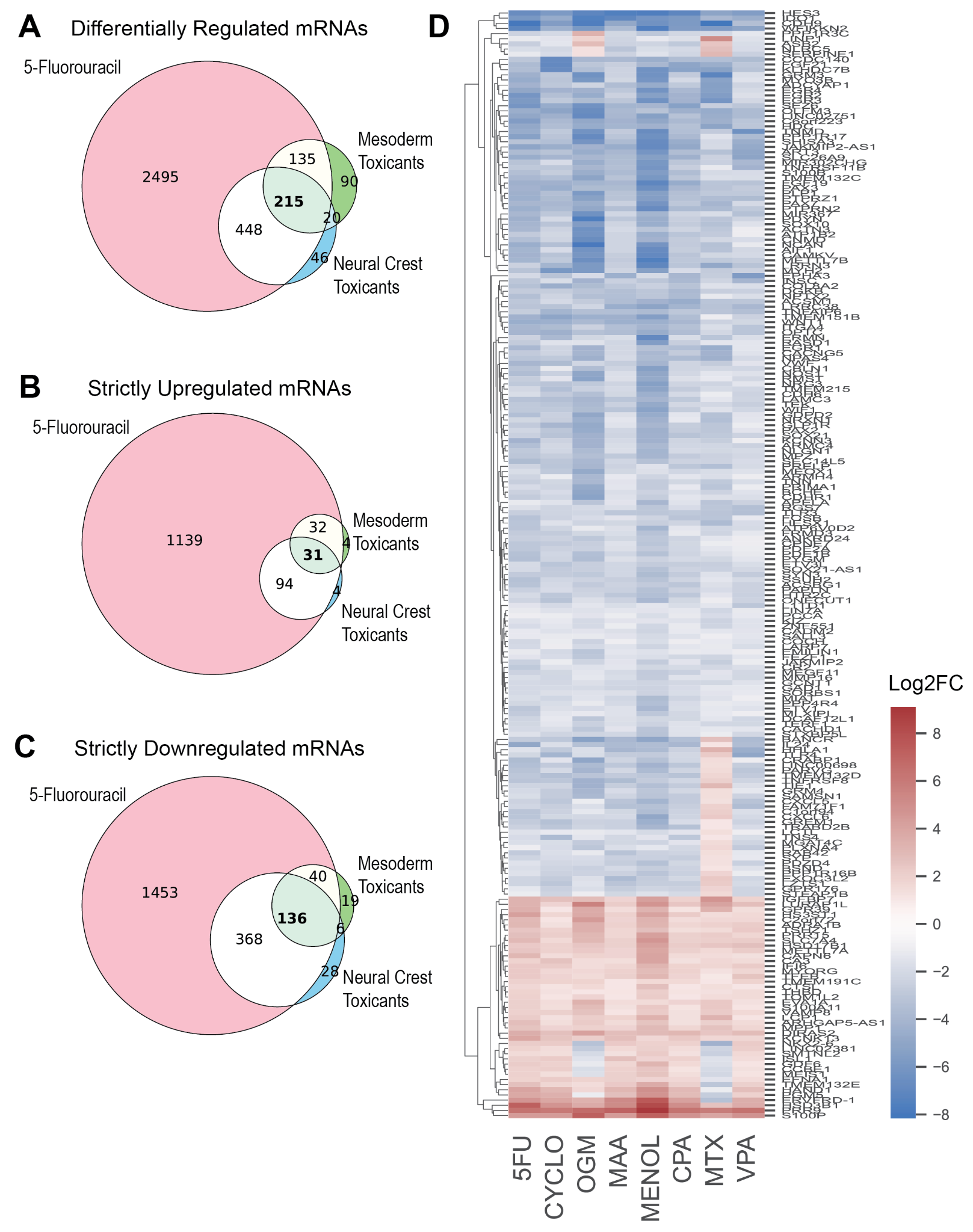


**Figure S6. Differential expression of mRNAs across eight developmental toxicants, excluding Hydrogen Peroxide (H2O2)**. Toxicants include 5-Fluorouracil (5FU), Cyclopamine (CYCLO), Ogremorphin (OGM), Methoxyacetic acid (MAA), Triadimenol (MENOL), Cyclophosphamide (CPA), Methotrexate (MTX), and Valproic acid (VPA). (A) Venn diagram showing overlap of consistently altered mRNAs (genes) (padj < 0.05) across toxicants and toxicant groups, identifying 215 mRNAs commonly regulated across all conditions with (B) showing those strictly upregulated and (C) showing those strictly downregulated. (D) Heatmap displaying the log₂ fold change of the 215 shared differentially regulated mRNAs across the eight treatments, illustrating consistent up- and downregulation patterns among neural crest toxicants, mesoderm toxicants, and 5-Fluorouracil.
