# Supplementary material for: MicroRNA network regulation of developmental bone toxicity in a human embryonic stem cell osteogenic model
Source: NAM J. 2026 Jul 2;2:100108. doi: 10.1016/j.namjnl.2026.100108 (PMC13355823; doi:10.1016/j.namjnl.2026.100108)
Supplement: Supplementary file 2 [file mmc2.docx]

**Supplemental Table S1.** Primer sequences.

|  | Forward | Reverse |
| --- | --- | --- |
| *CYR61* | 5’-CAACCCTTTACAAGGCCAGA-3’ | 5’-TTCAGGCTGCTGTACACTGG-3’ |
| *DLX5* | 5’-ACGCTAGCTCCTACCACCAG-3’ | 5’-TTTGCCATTCACCATTCTCA-3’ |
| *DLX6* | 5’-GCTGAAGCAGGGCAGTAATC-3’ | 5’-CTGTGGTCTCTGCATCGTGT-3’ |
| *FGF13* | 5’-GAAGTCTGCTGCAGGGGTAG-3’ | 5’-GGTGCAAGCTATTGGTGGAT-3’ |
| *FOXO1* | 5'-TAGCATAAACCTGGGCCCAA-3’ | 5’-ACCAGCTTGCCCATTACTCT-3’ |
| *FOXO3* | 5’-CGCACCAATTCTAACGCCAG-3’ | 5’-CTGCCATATCAGTCAGCCGT-3’ |
| *MIER1* | 5’-TTCTCCCATTCCTTGTCTGC-3’ | 5’-GGGACCAAGAACCACTGAAA-3’ |
| *MIXL1* | 5’-AGCTGCTGGAGCTCGTCTT-3’ | 5’-CGCCTGTTCTGGAACCATAC-3’ |
| *PAX7* | 5’-CACTGTGACCGAAGCACTGT-3’ | 5’-GTCAGGTTCCGACTCCACAT-3’ |
| *RUNX2* | 5’-TTACTTACACCCCGCCAGTC-3’ | 5’-TATGGAGTGCTGCTGGTCTG-3’ |
| *SNAI2* | 5’-CAGACCCTGGTTGCTTCAAG-3’ | 5’-GAGCCCTCAGATTTGACCTG-3’ |
| *SOX10* | 5’-CCTTCATGGTGTGGGCTC-3’ | 5’-CGCTTGTCACTTTCGTTCAG-3’ |
| *SOX9* | 5’-GTACCCGCACTTGCACAAC-3’ | 5’-GCGGCTGGTACTTGTAATCC-3’ |
| *TBX6* | 5’-AGAACCGGGAGCTATGGAAG-3’ | 5’-ACCGGAATCACATCCAGAAG-3’ |
| *TFAP2α* | 5’-GATCCTCGCAGGGACTACAG-3’ | 5’-TACCCGGGTCTTCTACATGC-3’ |
| *GAPDH* | 5’-GAGTCAACGGATTTGGTCGT-3’ | 5’-TTGATTTTGGAGGGATCTCG-3’. |

**Supplemental Table S2.** Consolidated summary of miRNAs implicated in osteogenic regulation, bone differentiation, and skeletal disorders.

| **miRNA** | **Direction** | **Bone-Related Targets** | **Outcome/Bone Disorder Association** | **Mechanistic Interpretation** | **Evidence Type** | **References** |
| --- | --- | --- | --- | --- | --- | --- |
| miR-145-5p | Down | SP7 (OSX), CBFB (Runx2 complex) | Delayed ossification, low bone mass | Alters RUNX2/SP7 regulatory balance, impairing osteoblast lineage commitment | In vitro, in vivo | Fukuda et al., 2015; Mazziotta et al., 2021 |
| miR-15b-5p | Down | SMURF1 (BMP/Runx2 axis) | Osteopenia, poor mineralization | Increased SMURF1 destabilizes RUNX2 and weakens BMP-driven osteogenesis | In vitro | Huang et al., 2022; Vimalraj et al., 2014; Wildman et al., 2019 |
| miR-1296-5p | Down | Predicted Wnt/β-catenin regulators | Reduced bone formation potential | Likely weakens canonical Wnt signaling needed for osteoprogenitor expansion | In silico (miRTarBase/TarBase/TargetScan) | Huang et al., 2022; Karagkouni et al., 2018 |
| miR-532-3p | Down | ETS1 | Reduced osteoblast survival | Increased apoptosis limits osteogenic cell pool during differentiation | In vitro | Fan et al., 2020; Huang et al., 2022, 2021; Mazziotta et al., 2021 |
| miR-522-5p | Down | Predicted MAPK/Wnt pathway nodes | Delayed osteogenic progression | Disrupts MAPK-mediated proliferation–differentiation transitions | In silico (miRTarBase/TarBase/TargetScan) | Huang et al., 2022; Karagkouni et al., 2018 |
| miR-106a-3p | Down | Predicted BMP/Wnt pathway regulators | Impaired osteoblast maturation | Reduces responsiveness to BMP signals required for late differentiation | In silico (miRTarBase/TarBase/TargetScan) | Huang et al., 2022; Karagkouni et al., 2018 |
| miR-302d-5p | Down | Predicted TGF-β/BMP regulators | Skeletal hypoplasia | Persistence of pluripotency signaling blocks mesenchymal commitment | In silico (miRTarBase/TarBase/TargetScan) | Huang et al., 2022; Karagkouni et al., 2018 |
| miR-454-5p | Down | Predicted Wnt/BMP pathway regulators | Delayed ossification | Reduced WNT9A signaling diminishes osteogenic Wnt activity | In silico (miRTarBase/TarBase/TargetScan) | Huang et al., 2022; Karagkouni et al., 2018 |
| miR-3940-3p | Down | Predicted osteogenic signaling regulators | Undetermined skeletal risk | Identified as dysregulated candidate; functional bone role unresolved | In silico (miRTarBase/TarBase/TargetScan) | Huang et al., 2022; Karagkouni et al., 2018 |
| miR-522-3p | Down | Predicted osteogenic signaling regulators | Undetermined skeletal risk | Transcriptomic association with impaired differentiation | In silico (miRTarBase/TarBase/TargetScan) | Huang et al., 2022; Karagkouni et al., 2018 |
| miR-526b-5p | Down | Predicted osteogenic signaling regulators | Delayed osteogenesis | May impair transition from pluripotency to osteogenic lineage | In silico (miRTarBase/TarBase/TargetScan) | Huang et al., 2022; Karagkouni et al., 2018 |
| miR-339-5p | Down | DLX5 | Reduced bone density | Disrupts osteogenic transcriptional programs | In vitro, in vivo | Huang et al., 2022; Jiménez-Ortega et al., 2025; Wang et al., 2021 |
| miR-124-5p | Down | DLX5, DLX3 (osteogenic TFs) | Craniofacial and long-bone defects | Alters spatial and temporal activation of osteoblast genes | In vitro, in vivo | Qadir et al., 2015; Tang et al., 2019; Wildman et al., 2019 |
| miR-130b-5p | Down | PPARγ (lineage commitment) | Low bone mass, marrow adiposity | Shifts mesenchymal fate toward adipogenesis | In vitro, in vivo, clinical | Krishnan et al., 2023; Lin et al., 2025; Mazziotta et al., 2021 |
| miR-18a-3p | Down | Notch2 (Notch signaling) | Defective matrix formation | Perturbs TGF-β/SMAD signaling during osteoblast maturation | In vitro, in vivo | He et al., 2024; Huang et al., 2022; Lian et al., 2020; Xu et al., 2020 |
| miR-204-5p | Down | RUNX2 | Severe inhibition of bone formation | Dysregulation of RUNX2-centered osteogenic transcription | In vitro, in vivo, clinical | Huang et al., 2010; Sardar et al., 2026; Zhang et al., 2020 |
| miR-1180-3p | Down | NF-κB regulators | Osteoporosis susceptibility | May enhance inflammatory suppression of osteogenesis | In silico (miRTarBase/TarBase/TargetScan) | Mazziotta et al., 2021) |
| miR-4521 | Down | Unknown | Undetermined skeletal risk | Candidate miRNA linked to differentiation failure | Transcriptomic | Kuthethur et al., 2023; Mazziotta et al., 2021; Senfter et al., 2019 |
| miR-320d | Down | Predicted HOXA10/Runx2 network (miR-320 family) | Impaired mineralization | Part of miR-320 family suppressing osteoblast maturation | In silico (miRTarBase/TarBase/TargetScan) | Huang et al., 2022, 2016; Laxman et al., 2016; Wang et al., 2020 |
| miR-365a-3p | Down | HDAC4 | Growth-plate defects | Disrupts chondrocyte hypertrophy and endochondral ossification | In vitro, in vivo | Guan et al., 2011; Mazziotta et al., 2021; Yang et al., 2016 |
| miR-935 | Down | STAT1 | Reduced osteoblast proliferation and differentiation | miR-935 targets and negatively regulates STAT1 expression | In vitro, transcriptomic | Mazziotta et al., 2021; Zhang et al., 2021 |
| miR-92b-3p | Down | Pro-osteogenic signaling (context-dependent); ERK/JNK axis | Reduced osteoblast differentiation | Alters Notch-dependent fate decisions | In vitro, in vivo | Huang et al., 2022; Lu et al., 2023 |
| miR-320c | Down | Predicted HOXA10/Runx2 network (miR-320 family) | Osteopenia | Suppresses osteogenic gene expression | In silico (miRTarBase/TarBase/TargetScan) | Huang et al., 2022; S. Zhang et al., 2021 |
| miR-361-5p | Down | VEGFA (osteogenesis–angiogenesis coupling) | Poor fracture healing | Uncouples angiogenesis from osteogenesis | In vivo, in vitro | Fröhlich, 2019; Huang et al., 2022; Zhang et al., 2024 |
| miR-320a-3p | Down | HOXA10 (osteogenic program) | Reduced bone formation | Limits osteoblast functional maturation | In vitro | Huang et al., 2022, 2016; Laxman et al., 2016; |
| miR-320b | Down | Predicted HOXA10/Runx2 network (miR-320 family) | Reduced bone formation | Similar suppressive effects on RUNX2 signaling | In silico (miRTarBase/TarBase/TargetScan) | Huang et al., 2022; Laxman et al., 2016 |
| miR-185-3p | Up | BGN (BMP/Smad signaling) | Stress-related differentiation failure | Alters survival signaling under toxicant stress | In vitro, in vivo | Cui et al., 2019; Ghafouri-Fard et al., 2021; Huang et al., 2022 |
| miR-192-5p | Up | Predicted osteogenic regulators (requires validation) | Differentiation arrest | Promotes premature cell-cycle exit | In silico (miRTarBase/TarBase/TargetScan) | Fröhlich, 2019; Huang et al., 2022; Karagkouni et al., 2018 |
| miR-548e-3p | Up | Predicted osteogenic regulators (requires validation) | Undetermined skeletal risk | Candidate miRNA without defined bone targets | In silico (miRTarBase/TarBase/TargetScan) | Huang et al., 2022; Karagkouni et al., 2018 |
| miR-215-5p | Up | XIAP | Abnormal osteoblast maturation | Disrupts timing of differentiation | In vitro | Huang et al., 2022; Mazziotta et al., 2021; Yin et al., 2022 |
| miR-1255a | Up | Predicted osteogenic regulators (requires validation) | Undetermined skeletal risk | Transcriptomic association only | In silico (miRTarBase/TarBase/TargetScan) | Huang et al., 2022; Karagkouni et al., 2018 |
| miR-24-2-5p | Up | Predicted Wnt transcriptional regulators (TCF/LEF family) | Impaired osteogenic signaling | Alters Wnt transcriptional output | In silico / in vitro (context-dependent) | Huang et al., 2022; Mazziotta et al., 2021 |
| miR-21-5p | Up | SMAD7; PTEN (context-dependent) | Aberrant mineralization, OA-like changes | Skews PI3K–AKT and TGF-β signaling balance | In vitro, in vivo | Fröhlich, 2019; Li and Jiang, 2019; Mazziotta et al., 2021 |
| miR-34b-5p | Up | Cell cycle regulators / osteoblast differentiation program | Reduced osteoblast proliferation | Enforces premature cell-cycle suppression | In vitro, in vivo | Mazziotta et al., 2021; Wei et al., 2012; Wildman et al., 2019 |
| miR-34c-5p | Up | Cell cycle regulators / osteoblast differentiation program | Reduced osteoblast proliferation | Reinforces proliferation arrest during differentiation | In vitro, in vivo | Mazziotta et al., 2021; Wei et al., 2012; Wildman et al., 2019 |
| miR-125b-1-3p | Up | CBFB; SP7 (osterix) (context-dependent) | Delayed bone formation | Alters late osteoblast maturation | In vitro | Huang et al., 2022; Mazziotta et al., 2021; Mizuno et al., 2008 |
| miR-147b-3p | Up | NDUFA4/PI3K-AKT axis (osteoporotic fracture model); inflammation context | Inflammation-associated bone loss | Enhances inflammatory signaling detrimental to osteogenesis | In vitro | Guo et al., 2025; Huang et al., 2022; Jiang et al., 2021 |
| miR-148b-5p | Up | Pro-osteogenic program reported in MSCs (miR-148 family) | Dysregulated osteogenic programming | Alters DNA methylation of osteogenic genes | In vitro | Mazziotta et al., 2021; Mollazadeh et al., 2019; Zhao et al., 2013 |
| miR-330-5p | Up | Predicted extracellular matrix regulators (requires validation) | Abnormal matrix organization | Perturbs BMP-dependent extracellular matrix deposition | In silico (miRTarBase/TarBase/TargetScan) | Huang et al., 2022; Karagkouni et al., 2018 |

Note: Supplemental Table 2 summarizes bone-associated miRNAs identified from the 63 shared differentially expressed miRNAs shown in Figure 4. Included miRNAs exhibited significant enrichment (adjusted p < 0.05) for bone-related biological processes based on predicted and/or validated targets. miRNAs are organized by the strength of supporting evidence. For each miRNA, the table reports direction of dysregulation on day 7, key bone-related targets, associated skeletal outcomes, mechanistic interpretation, evidence level, and references.

**Supplemental Table S3.** Core miRNA–mRNA network interactions linking toxicant groups to bone-developmental gene regulation.

| **Toxicant group** | **Hub miRNAs** | **Principal bone-gene targets** | **Dominant pathway axes** | **Inverse coupling** | **Key note** |
| --- | --- | --- | --- | --- | --- |
| **Cytotoxic** (5FU) | miR-21-5p, miR-15b-5p, miR-92b-3p, miR-320, miR-145-5p | RUNX2, DLX5, SOX9, PTEN | BMP/TGF-β, apoptosis | Medium–High | Loss of miR-21-5p and gain of miR-145/320 mirrors mineralization loss. |
| **Neural-crest–targeting** (CYCLO, OGM, MAA, MENOL) | miR-320, miR-145-5p, miR-21-5p, miR-15b-5p, miR-92b-3p | DLX5/6, SOX10, TFAP2A, RUNX2 | SHH, BMP, Wnt/β-catenin | High | Crest-lineage suppression consistent with craniofacial skeletal vulnerability. |
| **Mesodermal-targeting** (CPA, MTX, VPA) | miR-21-5p, miR-15b-5p, miR-92b-3p, miR-320, miR-145-5p, miR-204-5p | RUNX2, DLX5, MIXL1, TBX6, SOX9 | DNA-damage, folate, HDAC/BMP | Very High | Strongest inverse coupling; VPA aligns with MTX/CPA through a shared high-centrality miRNA regulatory signature. |

Note: Supplemental Table 3 summarizes dominant hub miRNA–mRNA interactions derived from the network analyses shown in Figure 6 for cytotoxic (5FU), mesoderm-targeting, and neural crest–targeting toxicants. Hubs were defined by high degree centrality and recurrent targeting of multiple bone-related genes. Principal targets represent bone-related mRNAs regulated by multiple hub miRNAs within each group. Dominant pathway axes reflect functional annotation of these high-connectivity targets.

**Supplemental Table S4.** Degree centrality metrics for high-centrality miRNAs across toxicant groups.

| **miRNA** | **5FU** | **H2O2** | **Mesoderm Group** | **Neural Crest Group** | **Sum Degree** | **Avg Degree** |
| --- | --- | --- | --- | --- | --- | --- |
| miR-92b-3p | 0.537 | 0.742 | 0.653 | 0.620 | 2.552 | 0.638 |
| miR-15b-5p | 0.670 | 0.000 | 0.753 | 0.742 | 2.165 | 0.541 |
| miR-21-5p | 0.548 | 0.000 | 0.626 | 0.597 | 1.771 | 0.443 |
| miR-320a-3p | 0.399 | 0.000 | 0.500 | 0.469 | 1.369 | 0.342 |
| miR-320b | 0.326 | 0.000 | 0.446 | 0.409 | 1.182 | 0.295 |
| miR-145-5p | 0.190 | 0.455 | 0.208 | 0.185 | 1.038 | 0.260 |
| miR-148b-5p | 0.297 | 0.000 | 0.375 | 0.341 | 1.013 | 0.253 |
| miR-320c | 0.264 | 0.000 | 0.344 | 0.313 | 0.921 | 0.230 |
| miR-361-5p | 0.218 | 0.000 | 0.302 | 0.260 | 0.780 | 0.195 |
| miR-1296-5p | 0.103 | 0.273 | 0.151 | 0.139 | 0.665 | 0.166 |

Note: Degree centrality of the top 10 miRNAs identified across bipartite miRNA–mRNA networks constructed for four toxicant groups: 5-Fluorouracil (5FU), hydrogen peroxide (H2O2), mesoderm toxicants, and neural crest toxicants. Degree centrality represents the number of differentially expressed bone-related target genes connected to each miRNA, normalized to the total number of possible targets within each network. “Sum Degree” indicates cumulative connectivity across groups, and “Avg Degree” indicates mean centrality across networks**.**
